# Tissue and systemic inflammation in dystrophic epidermolysis bullosa: a systematic review and meta-analysis

**DOI:** 10.1186/s13023-025-04034-2

**Published:** 2025-09-23

**Authors:** Meropi Karakioulaki, Nana-Adjoa Kwarteng, Adriani Nikolakopoulou, Hanning Yang, Moritz Hess, Harald Binder, Kilian Eyerich, Cristina Has

**Affiliations:** 1 Department of Dermatology and Venereology, Faculty of Medicine and Medical Center, University Hospital Freiburg, Hauptstraße 7, 79104 Freiburg, Germany; 2 Institute of Medical Biometry and Statistics, Faculty of Medicine and Medical Center, University of Freiburg, Freiburg, Germany; 3 Laboratory of Hygiene, Social and Preventive Medicine and Medical Statistics, School of Medicine, Aristotle University of Thessaloniki, Thessaloniki, Greece

**Keywords:** Epidermolysis bullosa, Dystrophic epidermolysis bullosa, Tissue inflammation, Systemic inflammation, Cytokines, Autoantibodies, Network meta-analysis

## Abstract

**Background:**

Dystrophic epidermolysis bullosa (DEB) is a rare inherited skin disorder caused by mutations in the type VII collagen gene, leading to mucocutaneous blistering. Subsequent inflammation contributes to chronic wounds, scarring, and systemic complications. There is controversy over whether and how inflammation should be therapeutically targeted.

**Objective:**

This systematic review and meta-analysis aim to question tissue and systemic inflammation in DEB and identify inflammatory patterns and research gaps to improve patient management.

**Methods:**

A comprehensive search of MEDLINE via PubMed was conducted to identify studies examining “DEB and tissue or systemic inflammation”. Out of 663 studies identified, 37 met the inclusion criteria. Data for synthesis were extracted from studies assessing systemic inflammatory parameter levels in DEB patients. For outcomes with multiple available studies, we performed an exploratory network meta-analysis to compare the standardized mean difference in systemic inflammatory parameters across three patient groups: DEB patients, healthy controls, and patients with other types of epidermolysis bullosa (EB).

**Results:**

The point estimate results for IL-4, IL-6, tumor necrosis factor-alpha, C-reactive protein, immunoglobulin (Ig) A, IgG, and IgM, as well as anti-collagen VII, anti-BP230, anti-BP180 autoantibodies suggested elevated values in DEB patients compared to healthy patients or other EB patients. The estimated standardized mean differences showed lower values of interleukin (IL)-10, hemoglobin and serum albumin in DEB patients compared to controls or other EB patients.

**Conclusion:**

Current evidence is limited by small and heterogeneous patient cohorts, variability in study designs and reporting methods, and a predominant reliance on observational and retrospective descriptive studies. Well-designed clinical trials and prospective studies are necessary to further investigate inflammatory pathways and assess the efficacy of (targeted) anti-inflammatory therapies but are difficult to perform and cost-intensive. AI tools for small-data may support research in this field.

*PROSPERO Registration Number* CRD42024535352.

**Supplementary Information:**

The online version contains supplementary material available at 10.1186/s13023-025-04034-2.

## Background

Dystrophic epidermolysis bullosa (DEB) is a rare inherited skin blistering disease stemming from mutations in *COL7A1*, the gene responsible for encoding the alpha-1 chain of type VII collagen (C7). C7 is a fundamental component of the anchoring fibrils within the dermal–epidermal junction of the skin and mucous membranes [1]. These mutations may disrupt the adhesion of the epidermis to the dermis, causing profound mechanical fragility of the skin and mucosal tissues, rendering them susceptible to tearing even upon minimal mechanical stress, accompanied by local tissue inflammation. Inflammation in turn leads to a series of cutaneous and extracutaneous complications [2]. Depending on the type of *COL7A1* mutations (e.g. amino acid substitutions or premature termination codons), the phenotype ranges from localized and intermediate dominant or recessive DEB (RDEB) to severe RDEB.

Tissue and systemic inflammation are widely recognized as significant contributors to the pathogenesis of DEB. However, their specific patterns and position in the pathogenic chain of DEB have only been explored in small, heterogeneous cohorts. Moreover, controversy persists over whether and how systemic inflammation should be targeted therapeutically. The newly approved topical gene therapy with beremagene geperpavec [3] marks a significant advancement because it addresses the structural deficit of DEB. Since only a limited wound area is treated, its effect on the inflammatory component remains to be explored. This raises the question of whether additional systemic anti-inflammatory therapies may be necessary alongside topical gene therapy to reduce systemic complications—a question that remains unresolved.

On the other hand, anti-inflammatory therapies alone do not address the underlying genetic deficit in DEB individuals. However, they may still provide benefits by reducing inflammation at sites of blister formation. Clinical experience and transcriptomic profiling [4] suggest that patients may clinically improve under off-label use of immunomodulatory drugs, such as methotrexate, biologics, systemic steroids, or pre-conditioning before hematopoietic stem cell transplantation. If such therapeutic approaches prove beneficial, further classification will be required to determine the most appropriate therapy and identify which inflammatory pathways to target and at which time point.

A consolidated information resource on patterns in EB is crucial for developing prognostic models for the condition. Specifically, insights from the literature can serve as prior knowledge to address the typically sparse data available for rare diseases. This is particularly relevant for approaches leveraging artificial intelligence (AI), where prior knowledge can be transferred to augment or impute the limited data available to clinicians.

The objective of this systematic review and meta-analysis is to illuminate the patterns of tissue and systemic inflammation in DEB based on existing literature. We aim to clarify which systemic inflammation patterns are predominant in DEB compared to healthy controls and other epidermolysis bullosa (EB) types, while also identifying gaps in current knowledge.

## Methods

### Systematic review

Studies that examined “DEB and tissue or systemic inflammation” were eligible for inclusion in the systematic review. No restrictions were made regarding population characteristics, such as age, gender, or ethnicity. Eligible studies published from 01.01.2004–18.03.2024 were identified in MEDLINE via PubMed using free text terms (see Additional file 1, Table S1, and Additional file 2, Figure S1) and assessed by two independent reviewers. The risk of bias assessment was conducted for all studies using the *JBI Critical Appraisal Checklist for Cohort Studies* [5]. Additional file 1, Table S2 contains the exclusion criteria in detail and Table S3 the excluded studies.

### Metanalysis

To explore systemic inflammation in DEB, we extracted data from the existing literature. This enabled an assessment of whether inflammation parameters (e.g., cytokines, chemokines, leukocytes, CRP, autoantibodies, creatinine, albumin, hemoglobin) differ between DEB patients, healthy controls, or other EB types. Data were included from studies with at least two of the following groups: DEB, healthy controls, or other EB types. For case studies involving interventions, only baseline data- collected prior to the administration of the intervention, when reported- were extracted.

Means and standard deviations were extracted for meta-analysis, and median values were transformed to means, assuming normality [6]. We excluded studies without measures of central tendency, groups with only one participant, studies with a single patient group, and outcomes appearing in just one group.

Study and paper quality were assessed for comparability, but no evidence was downgraded. Publication bias and small-study effects were not evaluated, as no outcome included 10 or more studies. Different strategies were applied for quantitative exploration based on data availability (see Additional file 2, Figure S2). Single-study comparisons reporting medians were summarized descriptively, while those with means were analyzed using mean differences and pooled standard deviations.

According to the protocol, we initially planned to analyze continuous outcomes measured on the same scale as mean differences (MDs). However, initial analysis revealed differences in the underlying scales of raw scores and medians. To standardize values, they were converted to Cohen’s *d* and analyzed as standardized mean differences (SMDs) [7, 8]. Depending on the network structure, we synthesized SMDs using pairwise or network meta-analysis [9]. Network meta-analyses (NMA) were performed using random-effects models (RE-NMA) via the *netmeta* package [9]. We used the REML estimator to calculate network-wide between-study heterogeneity (τ^2^), assuming common heterogeneity across comparisons. 95% confidence and prediction intervals were generated for comparisons involving more than two studies. Networks were constructed differently than the conventional approach of NMA; we considered nodes to be patient groups: healthy controls (H), patients with other types of EB (PH), patients with DEB (P) and patients with DEB and other EB types grouped together (P/PH).

## Results: systematic review

**Fig. 1:**
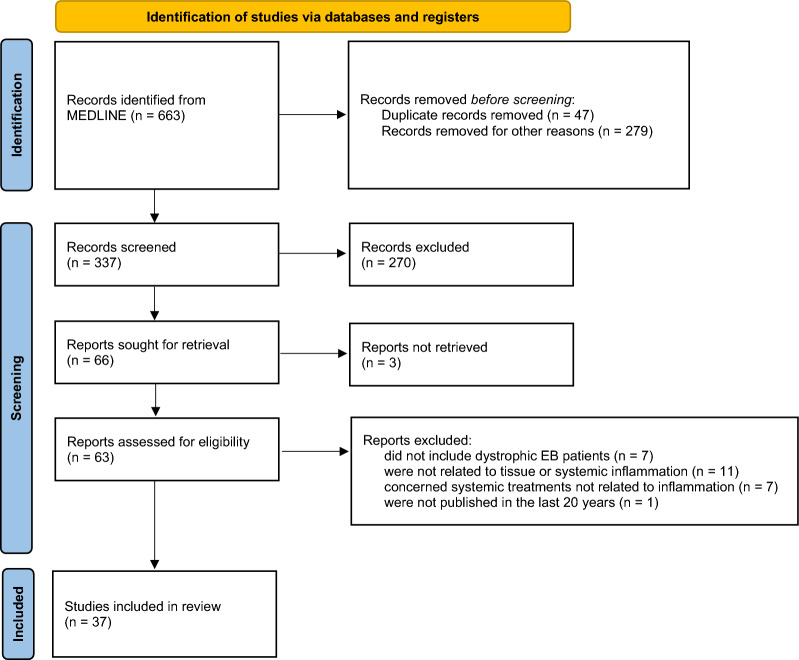
Prisma 2020 flow diagram for systematic reviews

A total of 663 studies were found, from which 37 were included in the systematic review (Fig. 1, Additional file 1, Table S4).

### Cutaneous tissue inflammation in DEB

Blisters and wounds are the main clinical manifestations of DEB. Wound healing is a complex and precisely regulated sequence of events: hemostasis, inflammation, proliferation, and remodeling [10]. In DEB, these stages do not resolve efficiently, with a particular dysregulation of or failure in resolving the inflammatory phase [11, 12] (see Additional file 1, Table S5).

A transcriptomic analysis investigated different expression profiles in skin biopsies of chronic wounds and intact skin from 6 RDEB and of intact skin from 6 controls [4]. RDEB wounds displayed aberrant cytokine-cytokine interactions, Toll-like receptor signaling and JAK-STAT signaling pathways, which may be the key activating events in RDEB wounds [4]. In the same study, interleukin 10 (IL-10), IL-20 and IL-6 signaling pathways were highlighted as potentially important dysregulated pathways in RDEB wounds [4].

In an immunohistochemistry study including skin samples from 11 patients with DEB, there was a significantly increased number of IL-17A positive cells per mm^2^ compared to normal skin [mean (SD): 217.0 (121.2) vs. 40.0 (10.0), p < 0.05] [13]. However, the number of IL-17A positive cells was lower than in psoriasis skin samples [360.0 (109.8)] [13].

In the study of Lee et al. investigating 11 RDEB patients and 11 healthy controls, the number of dermal IL-31 producing cells and the total fluorescence intensity of IL-31 were significantly increased in the skin of RDEB patents, when compared to controls (p < 0.001 and p < 0.001 respectively) [14]. These cells are associated with Th2 immunity [15] and other Th2-related markers, such as IL-4Ra ( +) and IL-13 ( +) cells, were higher in the skin of RDEB patients than in controls [14]. Moreover, the expression of serum thymic stromal lymphopoietin (TSLP) and periostin were significantly increased in RDEB skin when compared to controls [14].

An mRNA and protein analysis of skin samples from 12 severe RDEB patients and 12 healthy donors (healthy skin and burn scars) indicated that mRNA levels of IL-1β and IL-6 were significantly upregulated in RDEB scars compared to healthy skin and intact RDEB skin [16]. IL-6 expression was also increased in scars from RDEB patients compared to scars of healthy donors [16]. Keratinocytes and dermal fibroblasts derived from lesional skin from RDEB patients showed abundant expression of IL-6 and serum amyloid A (SAA), while in healthy skin, IL-6 expression was confined to the granular and spinous layers of the epidermis and SAA expression was completely absent [17].

Identical *COL7A1* mutations may often result in inter- and intra-familial disease variability, suggesting that there might be additional modifiers that contribute to the course of RDEB [18, 19]. A genome-wide expression analysis in fibroblasts deriving from a monozygotic twin pair with RDEB with different phenotypic manifestations but similar amounts of C7 expression showed a different expression of genes associated with TGF-β pathway [20]. Fibroblasts from the less affected twin expressed decorin, a skin matrix component with anti-fibrotic properties [20]. On the other hand, fibroblasts from the more affected twin manifested a profibrotic and contractile phenotype characterized by enhanced α-smooth muscle actin and plasminogen activator inhibitor 1 expression, collagen I release and collagen lattice contraction [20]. These cells also produced increased amounts of proinflammatory cytokines (IL-6 and monocyte chemoattractant protein I, MCP-I/CCL2) and both canonical (Smads) and non-canonical (MAPKs) TGF-β pathways were basally more activated, when compared to the less affected sibling [20].

### Systemic inflammation in DEB

Serum cytokine levels are often elevated in patients with inflammatory diseases. However, these patients are frequently treated with corticosteroids, immunosuppressive agents, biologics, and anti-inflammatory drugs, which can suppress or normalize cytokine levels. In contrast, patients with DEB rarely receive such treatments. Nonetheless, numerous studies have demonstrated elevated serum levels of various cytokines in DEB patients, contributing to chronic systemic inflammation and worsening of the clinical course, due to the development of systemic extracutaneous manifestations (see Additional file 1, Table S6).

In a pediatric cohort of 29 patients with JEB (n = 5) and DEB (n = 24), most patients had elevated CRP levels, particularly those with severe RDEB [21]. IL-6 emerged as the most consistently and highly aberrant cytokine, dominating systemic inflammation in these patients [21]. These findings were validated in a cohort of 42 patients (14 with JEB, 28 with DEB) [21]. IL-6 levels correlated with wound body surface area in both cohorts [21]. TGF-β was only marginally elevated in patients with severe RDEB, while TNF-a, interferon-gamma (IFN-γ), and IL-1β levels varied inconsistently [21]. Moreover, in the same study, patients reporting itch showed elevations in Th2 inflammation markers (IgE, TSLP, IL-4, and/or IL-31) [21]. Similarly, another study of 5 EB patients (4 DEB, 1 JEB) demonstrated that all patient sera had increased IL-6 levels [22].

Serum levels of IL-6 and IL-10 were also measured in 13 patients with RDEB, 10 with EBS and 18 healthy controls [23]. IL-6 was statistically higher in patients with RDEB compared to patients with EBS and healthy subjects (p < 0.001), while IL-10 was statistically lower in patients with RDEB than in EBS patients and healthy subjects (p = 0.002) [23]. Moreover, the study demonstrated that IL-6/IL-10 ratio > 5.6 has a good diagnostic accuracy to identify patients with the highest severity of disease [23].

A case report of two patients with generalized RDEB indicated a persistent elevation of IL-6 and SAA in these patients [24]. SAA is an acute-phase protein produced mainly in the liver and its persistent high concentration in serum is considered a prerequisite for the development of systemic amyloidosis [25]. IL-6 induces expression of SAA and suppresses albumin production in the liver [26]. This, together with the leakage of albumin in the skin ulcers, results in hypoalbuminemia, leading to the compensating production of gamma globulin, including IgA [24]. This might be associated with the development of secondary amyloidosis and IgA nephropathy [24]. Similarly, in a study of 7 RDEB patients, 33 psoriasis patients, 32 atopic dermatitis patients and 30 healthy volunteers, RDEB patients demonstrated significantly higher levels of IL-6 and SAA compared to the other patient groups [17].

An analysis of serum samples from 25 DEB patients indicated that IL-1β, IL-6, TNF-β and IFN-γ serum levels were significantly increased compared to 9 healthy controls [27]. IL-2 levels were also significantly increased in DEB patients compared to 15 EBS patients [27].

Another study compared serum levels of cytokines in 42 EB patients (13 EBS, 22 DEB, 5 junctional EB- JEB, 2 Kindler EB) and 38 healthy controls. IL-1β, IL-2, IL-6, IL-10 and TNF-β, IFN-γ were significantly higher in all EB patients than in controls [28]. IL-6 was significantly higher in EB patients with higher Birmingham EB Severity scores than those with lower values [28].

Lee et al. included 11 RDEB patients and 11 healthy controls in their study and indicated that serum IL-31 levels are elevated in RDEB patients compared to healthy controls [14]. TSLP levels were also significantly elevated in DEB compared to controls [14].

In a retrospective study of 200 children with EB (157 with RDEB, 43 with JEB), elevated CRP levels were present in 77% of RDEB patients and 42% of JEB patients [29]. In RDEB patients, CRP levels increased with age and were significantly higher in those with severe subtypes compared to intermediate subtypes [29].

### Systemic anti-inflammatory treatments in DEB

Recent studies have explored several promising anti-inflammatory and immunomodulatory therapies for DEB (see Additional file 1, Table S7). Dupilumab, administered to two patients with DEB pruriginosa, led to marked clinical improvement—including reduced pruritus, fewer lesions, and improved quality of life—accompanied by immunological changes such as decreased Th2 cells and increased type VII collagen expression in the skin [30]. Similarly, losartan, given orally to seven RDEB patients for six weeks, resulted in subjective and objective clinical improvement, reduced dermal fibrosis, and increased vascularity and mast cell counts [31]. A transcriptomic profiling study identified methotrexate and statins among the top candidates capable of reversing the dysregulated gene signatures of RDEB, highlighting methotrexate in particular as a strong candidate for further clinical testing [4]. In a cohort of 12 DEB patients, JAK inhibitors (upadacitinib and baricitinib) significantly reduced itch, pain, erythema, and blister formation without serious side effects, suggesting their utility in breaking the itch-scratch-blister cycle [32]. A single case study of a patient with mild DEB and psoriatic arthritis showed that TNF-α blockade with etanercept led to rapid and sustained improvement of both joint symptoms and DEB-related skin manifestations [33]. Additionally, short-term systemic administration of G-CSF in 7 patients (6 RDEB, 1 DDEB) resulted in substantial reductions in wound surface area and blister count, without adverse effects [34]. Collectively, these findings underscore the therapeutic potential of anti-inflammatory and immune-targeted treatments in DEB, though most evidence is preliminary and warrants confirmation in larger controlled trials.

### Autoimmunity interplay with inflammation in DEB

Chronic systemic inflammation may result in a dysregulated immune system in EB patients. Additionally, skin damage and barrier defects caused by inherited structural anomalies may lead to continuous antigen exposure and to the production of autoantibodies against several proteins involved in keratinocyte adhesion or located at the dermal–epidermal junction. These proteins-including C7, desmoglein 1 (DSG1), desmoglein 3 (DSG3), collagen XVII/BP180, BP230, and laminin 332- are also targeted in autoimmune blistering diseases (AIBD). However, data regarding the pathogenicity of these autoantibodies are limited and inconsistent across existing studies (see Additional file 1, Table S8). This inconsistency may arise from various mechanisms leading to autoantibody generation, such as molecular mimicry, chronic impairment of the skin barrier, genetic variants of skin proteins with enhanced immunogenicity activating B-cells, and epitope spreading due to tissue damage and release of cryptic epitopes. Additionally, many factors can influence their pathogenicity, including T-cell activation (e.g., following PD1-inhibitor treatment), antigen specificity, immunoglobulin isotype, and glycosylation patterns of the Fc regions [35].

In a study of 5 JEB and 10 RDEB patients, 62 sera were analyzed for AIBD antibodies [36]. RDEB patients were more susceptible to developing such autoantibodies than JEB patients (70% vs 20% respectively) [36]. The pathogenicity of these autoantibodies was not investigated in this study [36].

In serum samples of 258 EB patients (19 EBS, 8 JEB, 231 DEB), 5.3% of EBS, 25.0% of JEB and 23.4% of DEB patients demonstrated high BP180 autoantibody titers [37]. The titers correlated negatively with C7 skin expression and positively with disease severity [37]. The prevalence of anti-BP180 autoantibodies was found 42 times higher in this EB cohort than in the general population [37].

In another study of 17 RDEB and 10 EBS patients, RDEB patients had statistically higher mean concentrations of C7, BP180 and BP230 autoantibodies [23]. The BEBS score was correlated with the anti-skin autoantibodies titers; however, their pathogenic role was unclear in their study and their presence was interpreted as an epiphenomenon [23].

In a study of 42 EB patients (13 EBS, 22 DEB, 5 JEB, 2 Kindler EB) and 38 healthy controls anti-skin antibodies were significantly higher in EB patients than in controls [28]. Anti-skin antibodies were significantly higher in patients with RDEB than in those with other types of EB, in generalized than localized EB and in patients with higher BEBS scores than in those with lower values [28].

## Results: meta-analysis

**Fig. 2:**
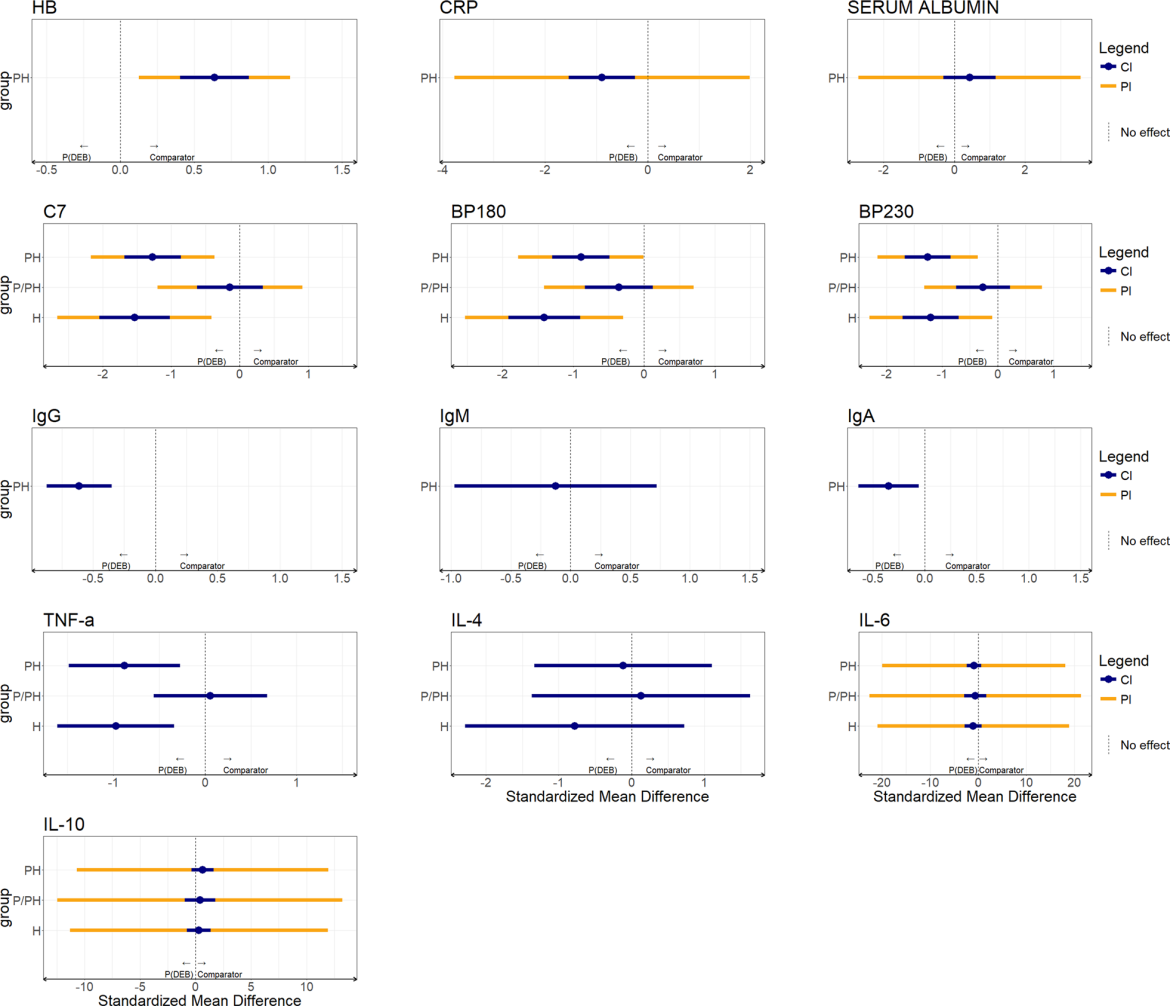
Summary estimates for comparisons with multiple sources of evidence. Values less than 0 indicate lower levels of the marker in the comparator group when compared to DEB. Values greater than 0 indicate higher levels of the marker in comparator group when compared to DEB. HB: hemoglobin, CRP: C-reactive protein, C7: collagen type VII autoantibodies, BP180: anti-BP180 autoantibodies, BP230: anti-BP230 autoantibodies, IgG: immunoglobulin G, IgM: immunoglobulin M, IgA: immunoglobulin A, TNF-a: tumor necrosis factor alpha, IL-4: interleukin 4, IL-6: interleukin 6, IL-10: interleukin 10, P: patients with dystrophic epidermolysis bullosa, PH: patients with other forms of epidermolysis bullosa, H: healthy controls, CI: 95% confidence intervals for the standardized mean difference estimates, PI: 95% prediction intervals for the standardized mean differences using the z-distribution, which were generated for comparisons involving more than two studies using the t-distribution

The initial dataset included 39 inflammation markers across four groups (H, PH, P and P/PH) from 23 studies (see Additional file 1, Table S9). The final dataset included 32 inflammation markers across the four groups from 11 studies (see Additional file 2, Figure S1) and three types of population comparisons were conducted (see Additional file 2, Figure S3). For single-study comparisons reporting medians, only descriptive summaries were possible (see Additional file 1, Table S10). Among the 11 inflammation markers containing data from single studies reporting means, IgE, TSLP, and leukocyte levels were higher in DEB patients than in other EB patients (Table 1). For markers with data from multiple studies, heterogeneity variance ranged from 0 to 1.64. For the 13 inflammation markers with evidence from multiple studies, NMA results are given in Fig. 2 and Additional file 1, Table S11. IL-6 values were, on average, lower in healthy controls [− 1.028 SMDs (95%CI − 2.793; 0.738)] and patients with other types of EB [− 0.894 SMDs (95%CI − 2.406; 0.617)] when compared to patients with DEB. In contrast, when compared to patients with DEB, IL-10 values were higher in healthy controls [0.313 SMDs (95%CI − 0.762;1.388)] and patients with other types of EB [0.642 SMDs (95%CI − 0.359;1.642)]. IgG, IgA, and CRP were higher in DEB compared to other EB patients, while autoantibodies (C7, BP180, BP230) were higher in DEB compared with both controls and other EB types (Fig. 2).

**Table 1:** Mean differences of the 11 systemic inflammatory markers from single studies reporting means

Inflammation Marker	Units	Reference group	Comparator group	Number of available studies	Reference	Mean Difference	95% CI
CCL19	pg/mL	PH	H	1	[41]	180.00	(– 314.41; 674.41)
CCL21	pg/mL	PH	H	1	[41]	872.70	(341.64; 1403.76)
CCL27	pg/mL	PH	H	1	[41]	– 47.20	(– 152.26; 57.86)
CCL28	pg/mL	PH	H	1	[41]	– 40.90	(– 1221.27; 1139.47)
CXCL12	pg/mL	PH	H	1	[41]	– 30.40	(– 44.87; – 15.93)
HMGB1	ng/mL	PH	H	1	[41]	– 8.90	(– 14.86; – 2.94)
Leukocytes	cells/mm^3^	P	PH	1	[29]	– 1.60	(– 2.55; – 0.65)
Creatinine	mg/dL	P	PH	1	[45]	0.18	(0.11; 0.25)
IgE	kU/L	P	PH	1	[21]	– 248.81	(– 480.51; – 17.11)
TGF-β	ng/dL	P	PH	1	[21]	4.75	(– 12.51; 22.01)
TSLP	pg/mL	P	PH	1	[21]	– 14.90	(– 34.7; 4.9)

CCL: Chemokine (C–C motif) ligand, CXCL: Chemokine (C-X-C motif) ligand, HMGB1: High mobility group box 1, TGF-β: Transforming growth factor β, TSLP: serum thymic stromal lymphopoietin, IgE: Immunoglobulin E, CI: confidence interval. Summary Estimates describe mean differences between comparators and reference groups in inflammatory markers with single study evidence. Networks were constructed differently than the conventional approach of NMA; we considered nodes to be patient groups: P: patients with dystrophic epidermolysis bullosa, PH: patients with other types of epidermolysis bullosa, H: healthy controls

## Conclusions

Our systematic review and meta-analysis indicate that tissue and systemic inflammation are involved in the pathogenic cascade of DEB, with inflammatory markers consistently elevated in DEB patients across studies. However, the available evidence does not yet support a coherent or unified model of inflammatory patterns in DEB. Moreover, the rarity of the disease contributes to significant study limitations and persistent knowledge gaps (see Additional file 1, Table S12).

Most studies are observational, retrospective, or descriptive, with no mechanistic explanation of the observed abnormalities. The existing studies are few, heterogeneous, and limited in sample size (n = 1–28), with most inflammation parameters covered by only 1 to 4 studies. High heterogeneity (τ^2^ = 0–1.64) observed across studies, reflects variability in study populations, study designs, outcome measurements and reporting methods. This heterogeneity underscores the inconclusive nature of the current evidence and highlights the need for more targeted and standardized research in this area. Many studies combine mean and median values, introducing estimation bias. Additionally, studies often group all EB types together [11, 27, 28], complicating DEB-specific conclusions. Further, DEB subtypes (pruriginosa, intermediate, localized, severe) are not consistently analyzed, adding further confounding factors. The majority of the included studies focused predominantly on patients with RDEB, with limited data available on the dominant DEB (DDEB) subtype. Due to the scarcity and heterogeneity of subtype-specific data, it was not possible to perform separate meta-analysis for RDEB and DDEB. Therefore, while the findings highlight a clear inflammatory profile in DEB patients, caution should be exercised when generalizing these results to all DEB subtypes. Future studies with larger cohorts and detailed subtype stratification are essential to better understand the differences in inflammatory responses between RDEB and DDEB, which may have important implications for personalized therapeutic approaches.

**Fig. 3:**
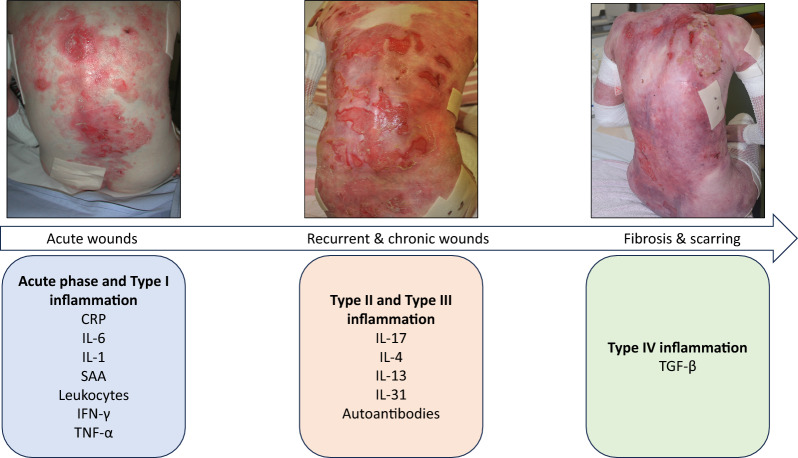
Proposed model of cutaneous inflammation in DEB [38], illustrating its progression across disease stages. In the acute phase, inflammation is driven by acute-phase reactants (CRP, IL-6, SAA) and type I cytokines (IFN-γ, TNF-α), triggered by acute blisters, wounds, and bacterial colonization. As the disease advances and wounds become recurrent or chronic, type II inflammation emerges, characterized by blisters, eczematous lesions, and elevated IL-4, IL-13, IL-31, and autoantibodies. Concurrently, type III inflammation arises, involving impaired barrier homeostasis, delayed wound healing, and IL-17 activity. In advanced chronic stages, tissue destruction exacerbates type IV inflammation, marked by fibrosis and scarring mediated by TGF-β, compounding previous inflammatory pathways. These overlapping processes contribute to systemic inflammation and drive the pathogenesis of aggressive cutaneous squamous cell carcinomas in DEB. Representative images depict the progression of skin changes on the back of a male RDEB patient at ages 8, 12, and 23 years. CRP: C-reactive protein, TNF-a: tumor necrosis factor alpha, IL: interleukin, TGF-β: Transforming growth factor β, SAA: serum amyloid A

It is important to note that inflammatory molecules are not the root cause of DEB; the disease is initiated by C7 deficiency, leading to large wound areas, wound healing failure, and subsequent persistent cytokine overexpression. A model of inflammation patterns in DEB [38] is proposed in Fig. 3. This model hypothesizes that acute inflammation in early-stage DEB is driven by acute-phase reactants (e.g., CRP, IL-6, SAA) and type I cytokines (e.g., IFN-γ, TNF-α) in response to blisters, wounds, and bacterial colonization. Over time, chronic wounds foster type II inflammation (e.g., IL-4, IL-13, IL-31, autoantibodies), accompanied by type III inflammation (e.g., IL-17) due to impaired barrier function and delayed wound healing. Advanced chronic stages may feature type IV inflammation, marked by TGF-β-mediated fibrosis and scarring. This model highlights the need for targeted interventions across the disease trajectories to improve outcomes for DEB patients.

Large-scale prospective multicenter studies on the natural history of DEB inflammation are needed. However, such studies require complex designs, significant funding, and decades to complete. Ethical concerns further complicate interventional trials, as placing patients in placebo groups is challenging given the severity of the disease. Still, comparing the efficacy of anti-inflammatory drugs, cytokine inhibitors, and dual-agent therapies is critical to addressing unmet clinical needs.

There is also an unmet need for longitudinal studies investigating bacterial colonization and its impact on wound resolution, as well as the natural progression of inflammation in DEB. Key unanswered questions include whether there is a critical window for treating inflammation (e.g., before age 10) and at what point inflammation causes irreversible systemic complications.

AI-powered tools for small-data [39] may offer new potential for studying complex rare and heterogeneous diseases like DEB, especially given the logistical challenges DEB patients face in participating in clinical trials. AI can predict missing data and accelerate insights while longitudinal studies are underway (see Additional file 1, Table S12).

## Supplementary Information

Additional file 1.

Additional file 2.

## Data Availability

The data that support the findings of this study are available from the corresponding author upon reasonable request.

## Abbreviations

95% CI: 95% Confidence interval
AI: Artificial intelligence
AIBD: Autoimmune blistering diseases
BDASI: Epidermolysis Bullosa Disease Activity and Scaring Index
BEBS: Birmingham EB Severity score
C7: Type VII collagen
CCL: Chemokine (C–C motif) ligand
CCL27: Cutaneous T-cell attracting chemokine
CDLQI: Children’s Dermatology Life Quality Index,
CRP: C-reactive protein
CXCL: Chemokine (C–X–C motif) ligand
DDEB: Dominant dystrophic epidermolysis bullosa
DEB: Dystrophic epidermolysis bullosa
DSG1: Desmoglein 1
DSG3: Desmoglein 3
EB-QoL: Epidermolysis bullosa quality of life questionnaire,
G-CSF: Granulocyte-colony stimulating factor
HB: Hemoglobin
HMGB1: High mobility group box 1
IFN-γ: Interferon-gamma
Ig: Immunoglobulin
IL: Interleukin
JAK: Janus kinase
MDs: Mean differences
NMA: Network meta-analysis
RDEB: Recessive dystrophic epidermolysis bullosa
RE NMA: Random effects network meta-analysis
SAA: Serum amyloid A
SMDs: Standardized mean differences
SF: Supplementary Figure
ST: Supplementary Table
TGF-β: Transforming growth factor β
TNF-a: Tumor necrosis factor alpha
TNF-β: Tumor necrosis factor beta
TSLP: Serum thymic stromal lymphopoietin
VAS: Visual Analog Scale
