# Supplementary material for: Tissue and systemic inflammation in dystrophic epidermolysis bullosa: a systematic review and meta-analysis
Source: Orphanet J Rare Dis. 2025 Sep 23;20:479. doi: 10.1186/s13023-025-04034-2 (PMC12455789; doi:10.1186/s13023-025-04034-2)
Supplement: Supplementary file 1 — Additional file 1. [file 13023_2025_4034_MOESM1_ESM.docx]

**Supplementary Tables**

**Table S1**. Studies included in the systematic review.

| **Database** | **Search Term** | **Date of Search** | **Total studies found** | **Studies included** |
| --- | --- | --- | --- | --- |
| Medline | Epidermolysis bullosa AND cytokines | 14.01.2024 | 148 | 20 |
|  | Epidermolysis bullosa AND hemoglobin | 30.01.2024 | 13 | 3 |
|  | Epidermolysis bullosa AND anemia | 02.02.2024 | 100 | 3 |
|  | Epidermolysis bullosa AND growth | 14.02.2024 | 304 | 3 |
|  | Epidermolysis bullosa AND autoantibodies NOT acquisita | 14.03.2024 | 66 | 3 |
|  | Epidermolysis bullosa AND inflammation | 18.03.2024 | 32 | 5 |

**Table S2**. Exclusion criteria of the systematic review.

| We excluded studies that:   - were systematic reviews or expert opinions, - did not include dystrophic EB patients, - were animal and cell studies, experimental gene therapies in cell models, - concerned acquired autoimmune blistering diseases, such as epidermolysis bullosa acquisita, bullous pemphigoid, pemphigus vulgaris, - concerned other genodermatoses, - were not related to inflammation, - concerned operative procedures or anesthetic protocols, - concerned systemic treatments not related to inflammation, - concerned exclusively squamous cell carcinoma in EB patients, - were not written in English, - were not published in the last 20 years, in order to make sure the genetic testing was performed and the diagnosis was reliable. |
| --- |

**Table S3**: Studies excluded from the systematic review according to the exclusion criteria.

| **Did not include dystrophic EB patients (n=7)** |
| --- |
| 1. Roth W, Reuter U, Wohlenberg C, Bruckner-Tuderman L, Magin TM. Cytokines as genetic modifiers in K5-/- mice and in human epidermolysis bullosa simplex. Hum Mutat. 2009;30(5):832-41. 2. Lettner T, Lang R, Klausegger A, Hainzl S, Bauer JW, Wally V. MMP-9 and CXCL8/IL-8 are potential therapeutic targets in epidermolysis bullosa simplex. PLoS One. 2013;8(7):e70123. 3. Castela E, Tulic MK, Rozieres A, Bourrat E, Nicolas JF, Kanitakis J, et al. Epidermolysis bullosa simplex generalized severe induces a T helper 17 response and is improved by apremilast treatment. Br J Dermatol. 2019;180(2):357-64. 4. Kim M, Jain S, Harris AG, Murrell DF. Colchicine may assist in reducing granulation tissue in junctional epidermolysis bullosa. Int J Womens Dermatol. 2016;2(2):56-9. 5. Yuen WY, Duipmans JC, Molenbuur B, Herpertz I, Mandema JM, Jonkman MF. Long-term follow-up of patients with Herlitz-type junctional epidermolysis bullosa. Br J Dermatol. 2012;167(2):374-82. 6. Filoni A, Bonamonte D, Cicco G, Panza R, Bisceglie V, Laforgia N. Nutritional impairment of neonates with epidermolysis bullosa: a retrospective study. Ital J Dermatol Venerol. 2022;157(4):330-4. 7. Mellerio JE, Kiritsi D, Marinkovich MP, Haro NR, Badger K, Arora M, et al. Mapping the burden of severe forms of epidermolysis bullosa - Implications for patient management. JAAD Int. 2023;11:224-32. |
| **Were not related to tissue or systemic inflammation (n=11)** |
| 1. Dehghani S, Akbarzadeh Pasha B, Karimi A, Afshin A. Severe hyponatremia in an infant with epidermolysis bullosa: a case report. J Med Case Rep. 2022;16(1):358.  2. Bruckner AL, Bedocs LA, Keiser E, Tang JY, Doernbrack C, Arbuckle HA, et al. Correlates of low bone mass in children with generalized forms of epidermolysis bullosa. J Am Acad Dermatol. 2011;65(5):1001-9.  3. Tarango C, Quinn CT, Augsburger B, Lucky AW. Iron status and burden of anemia in children with recessive dystrophic epidermolysis bullosa. Pediatr Dermatol. 2023;40(2):288-93.  4. Eng VA, Solis DC, Gorell ES, Choi S, Nazaroff J, Li S, et al. Patient-reported outcomes and quality of life in recessive dystrophic epidermolysis bullosa: A global cross-sectional survey. J Am Acad Dermatol. 2021;85(5):1161-7.  5. Yavuz Y, An I, Yazmaci B, Akkus Z, Ortac H. Evaluation of Clinical and Oral Findings in Patients with Epidermolysis bullosa. Medicina (Kaunas). 2023;59(7).  6. Zidorio APC, Leao DOD, De Carvalho KMB, Dutra ES. Nutritional outcomes in children with epidermolysis bullosa: long-term follow-up. Nutr Hosp. 2018;35(2):265-70.  7. Ingen-Housz-Oro S, Blanchet-Bardon C, Vrillat M, Dubertret L. Vitamin and trace metal levels in recessive dystrophic epidermolysis bullosa. J Eur Acad Dermatol Venereol. 2004;18(6):649-53.  8. Manjunath S, Mahajan R, De D, Handa S, Attri S, Behera BN, et al. The severity of malnutrition in children with epidermolysis bullosa correlates with disease severity. Sci Rep. 2021;11(1):16827.  9. Wasserman H, Dumenigo A, Hornung L, Augsburger B, Marathe K, Lucky AW. Prevalence of delayed puberty and low bone density in patients with epidermolysis bullosa: Insight from a large single center's experience. Pediatr Dermatol. 2023;40(1):100-6.  10. Colomb V, Bourdon-Lannoy E, Lambe C, Sauvat F, Hadj Rabia S, Teillac D, et al. Nutritional outcome in children with severe generalized recessive dystrophic epidermolysis bullosa: a short- and long-term evaluation of gastrostomy and enteral feeding. Br J Dermatol. 2012;166(2):354-61.  11. Kim KY, Namgung R, Lee SM, Kim SC, Eun HS, Park MS, et al. Nutritional outcomes in children with epidermolysis bullosa: the experiences of two centers in Korea. Yonsei Med J. 2014;55(1):264-9. |
| **Concerned systemic treatments not related to inflammation (n=7)** |
| 1. Tadini G, Pezzani L, Ghirardello S, Rebulla P, Esposito S, Mosca F. Cord blood platelet gel treatment of dystrophic recessive epidermolysis bullosa. BMJ Case Rep. 2015;2015.  2. Augsburger BD, Lucky AW, Marathe K, Tarango C. Enteral iron absorption in patients with recessive dystrophic epidermolysis bullosa. Pediatr Dermatol. 2020;37(5):817-20.  3. Kuo DJ, Bruckner AL, Jeng MR. Darbepoetin alfa and ferric gluconate ameliorate the anemia associated with recessive dystrophic epidermolysis bullosa. Pediatr Dermatol. 2006;23(6):580-5.  4. Alheggi A, McGrath JA, Hubbard L, Greenblatt DT, Mellerio JE. Treatment of multifactorial anaemia in adults with severe epidermolysis bullosa using intravenous ferric carboxymaltose: a single institution, observational, retrospective study. Br J Dermatol. 2023;188(2):306-7.  5. Fewtrell MS, Allgrove J, Gordon I, Brain C, Atherton D, Harper J, et al. Bone mineralization in children with epidermolysis bullosa. Br J Dermatol. 2006;154(5):959-62.  6. Woodley DT, Cogan J, Hou Y, Lyu C, Marinkovich MP, Keene D, et al. Gentamicin induces functional type VII collagen in recessive dystrophic epidermolysis bullosa patients. J Clin Invest. 2017;127(8):3028-38.  7. Hunjan MK, Bardhan A, Harper N, Balacco DL, Langman G, Suresh V, et al. IgA nephropathy in adults with epidermolysis bullosa. Clin Exp Dermatol. 2023;48(8):920-5. |
| **Were not published in the last 20 years (n=1)** |
| 1. Lechner-Gruskay D, Honig PJ, Pereira G, McKinney S. Nutritional and metabolic profile of children with epidermolysis bullosa. Pediatr Dermatol. 1988;5(1):22-7. |

**Table S4**: Studies included in the systematic review according to the inclusion criteria.

| 1. Annicchiarico G, Morgese MG, Esposito S, Lopalco G, Lattarulo M, Tampoia M, et al. Proinflammatory Cytokines and Antiskin Autoantibodies in Patients With Inherited Epidermolysis Bullosa. Medicine (Baltimore). 2015;94(42):e1528. 2. Esposito S, Guez S, Orenti A, Tadini G, Scuvera G, Corti L, et al. Autoimmunity and Cytokine Imbalance in Inherited Epidermolysis Bullosa. Int J Mol Sci. 2016;17(10). 3. Wu XG, Yan S, Jiang JQ, Zhou TT, Fang X, Yang H, et al. Successful treatment of epidermolysis bullosa pruriginosa by dupilumab. J Dermatol. 2023;50(6):837-42. 4. Alexeev V, Salas-Alanis JC, Palisson F, Mukhtarzada L, Fortuna G, Uitto J, et al. Pro-Inflammatory Chemokines and Cytokines Dominate the Blister Fluid Molecular Signature in Patients with Epidermolysis Bullosa and Affect Leukocyte and Stem Cell Migration. J Invest Dermatol. 2017;137(11):2298-308. 5. Matsushima Y, Mizutani K, Goto H, Nakanishi T, Kondo M, Habe K, et al. Emaciation, Congestive Heart Failure, and Systemic Amyloidosis in Severe Recessive Dystrophic Epidermolysis Bullosa: Possible Internal Complications Due to Skin-Derived Inflammatory Cytokines Derived from the Injured Skin. Dermatopathology (Basel). 2020;7(2):41-7 6. Lee SG, Kim SE, Jeong IH, Lee SE. Mechanism underlying pruritus in recessive dystrophic epidermolysis bullosa: Role of interleukin-31 from mast cells and macrophages. J Eur Acad Dermatol Venereol. 2023. 7. Pourani MR, Vahidnezhad H, Mansouri P, Youssefian L, Rakhshan A, Hajimoradi B, et al. Losartan treatment improves recessive dystrophic epidermolysis bullosa: A case series. Dermatol Ther. 2022;35(7):e15515 8. Breitenbach J, Gruber C, Klausegger A, Trost A, Bogner B, Reitsamer H, et al. Pseudosyndactyly - an inflammatory and fibrotic wound healing disorder in recessive dystrophic epidermolysis bullosa. J Dtsch Dermatol Ges. 2015;13(12):1257-66. 9. Aguado T, Garcia M, Garcia A, Ferrer-Mayorga G, Martinez-Santamaria L, Del Rio M, et al. Raloxifene and n-Acetylcysteine Ameliorate TGF-Signalling in Fibroblasts from Patients with Recessive Dominant Epidermolysis Bullosa. Cells. 2020;9(9) 10. Tampoia M, Abbracciavento L, Morrone M, Fumarulo R. IL-6/IL-10 Ratio as A Prognostic and Predictive Marker of the Severity of Inherited Epidermolysis Bullosa. Iran J Immunol. 2017;14(4):340-9. 11. Ujiie I, Fujita Y, Nakayama C, Matsumura W, Suzuki S, Shinkuma S, et al. Altered balance of epidermis-related chemokines in epidermolysis bullosa. J Dermatol Sci. 2017;86(1):37-45. 12. Onoufriadis A, Proudfoot LE, Ainali C, Torre D, Papanikolaou M, Rayinda T, et al. Transcriptomic profiling of recessive dystrophic epidermolysis bullosa wounded skin highlights drug repurposing opportunities to improve wound healing. Exp Dermatol. 2022;31(3):420-6. 13. Kwon IJ, Kim SE, Kim SC, Lee SE. Efficacy of oral JAK1 or JAK1/2 inhibitor for treating refractory pruritus in dystrophic epidermolysis bullosa: A retrospective case series. J Dermatol. 2024;51(3):441-7. 14. Gubinelli E, Angelo C, Pacifico V. A case of dystrophic epidermolysis bullosa improved with etanercept for concomitant psoriatic arthritis. Am J Clin Dermatol. 2010;11 Suppl 1:53-4. 15. Morizane S, Mizuno K, Takiguchi T, Ogita S, Nishida Y, Kawakami Y, et al. Persistent elevation of serum interleukin-6 and serum amyloid A levels in patients with recessive dystrophic epidermolysis bullosa. Eur J Dermatol. 2017;27(1):80-1. 16. Lettner T, Lang R, Bauer JW, Wally V. Increased levels of matrix metalloproteinase-9 and interleukin-8 in blister fluids of dystrophic and junctional epidermolysis bullosa patients. J Eur Acad Dermatol Venereol. 2015;29(2):396-8. 17. Fine JD, Manes B, Frangoul H. Systemic granulocyte colony-stimulating factor (G-CSF) enhances wound healing in dystrophic epidermolysis bullosa (DEB): Results of a pilot trial. J Am Acad Dermatol. 2015;73(1):56-61. 18. Kawakami Y, Oyama N, Ohtsuka M, Nakamura K, Kaneko F. Increased serum levels of interleukin-6, immunoglobulin and acute phase protein in patients with the severe clinical form of inherited epidermolysis bullosa. J Dermatol. 2005;32(6):503-5. 19. Lehman TD, Morgan TK, Lehman NL, Vogel H, McGuire JS. Recessive dystrophic epidermolysis bullosa associated with mesangioproliferative glomerulonephritis and multifocal necrotizing leucoencephalopathy of the pons. Br J Dermatol. 2004;151(6):1266-9 20. Odorisio T, Di Salvio M, Orecchia A, Di Zenzo G, Piccinni E, Cianfarani F, et al. Monozygotic twins discordant for recessive dystrophic epidermolysis bullosa phenotype highlight the role of TGF-beta signalling in modifying disease severity. Hum Mol Genet. 2014;23(15):3907-22. 21. Khanmohammadi S, Yousefzadeh R, Rashidan M, Hajibeglo A, Bekmaz K. Epidermolysis bullosa with clinical manifestations of sepsis and pneumonia: A case report. Int J Surg Case Rep. 2021;86:106258. 22. Reimer A, Hess M, Schwieger-Briel A, Kiritsi D, Schauer F, Schumann H, et al. Natural history of growth and anaemia in children with epidermolysis bullosa: a retrospective cohort study. Br J Dermatol. 2020;182(6):1437-48 23. Tsaqilah L, Diana IA, Gondokaryono SP, Effendi R, Suwarsa O, Gunawan H, et al. A Retrospective Study on the Clinical, Laboratory, and Nutritional Status of Pediatric Epidermolysis Bullosa in a Tertiary Referral Hospital in West Java, Indonesia. Clin Cosmet Investig Dermatol. 2023;16:1615-21. 24. Marchili MR, Spina G, Roversi M, Mascolo C, Pentimalli E, Corbeddu M, et al. Epidermolysis Bullosa in children: the central role of the pediatrician. Orphanet J Rare Dis. 2022;17(1):147. 25. Csikos M, Orosz Z, Bottlik G, Szocs H, Szalai Z, Rozgonyi Z, et al. Dystrophic epidermolysis bullosa complicated by cutaneous squamous cell carcinoma and pulmonary and renal amyloidosis. Clin Exp Dermatol. 2003;28(2):163-6. 26. Lara-Corrales I, Mellerio JE, Martinez AE, Green A, Lucky AW, Azizkhan RG, et al. Dilated cardiomyopathy in epidermolysis bullosa: a retrospective, multicenter study. Pediatr Dermatol. 2010;27(3):238-43 27. Rodari G, Guez S, Salera S, Ulivieri FM, Tadini G, Brena M, et al. A single-centre study on predictors and determinants of pubertal delay and growth impairment in Epidermolysis Bullosa. PLoS One. 2022;17(9):e0274072. 28. Rodari G, Guez S, Manzoni F, Chalouhi KK, Profka E, Bergamaschi S, et al. Birmingham epidermolysis severity score and vitamin D status are associated with low BMD in children with epidermolysis bullosa. Osteoporos Int. 2017;28(4):1385-92. 29. Fuentes I, Yubero MJ, Morande P, Varela C, Orostica K, Acevedo F, et al. Longitudinal study of wound healing status and bacterial colonisation of Staphylococcus aureus and Corynebacterium diphtheriae in epidermolysis bullosa patients. Int Wound J. 2023;20(3):774-83. 30. Bremer J, Pas HH, Diercks GFH, Meijer HJ, van der Molen SM, Nijenhuis AM, et al. Patients suffering from dystrophic epidermolysis bullosa are prone to developing autoantibodies against skin proteins: A longitudinal confirmational study. Exp Dermatol. 2024;33(2):e15035. 31. Lehr S, Felber F, Tantcheva-Poor I, Kessler C, Eming R, Nystrom A, et al. Occurrence of autoantibodies against skin proteins in patients with hereditary epidermolysis bullosa predisposes to development of autoimmune blistering disease. Front Immunol. 2022;13:945176. 32. Garcia-Espinosa L, Del Rosal T, Quintana L, Maseda R, Grasa C, Falces-Romero I, et al. Bloodstream Infection in Children With Epidermolysis Bullosa. Pediatr Infect Dis J. 2023;42(6):510-4. 33. Tampoia M, Bonamonte D, Filoni A, Garofalo L, Morgese MG, Brunetti L, et al. Prevalence of specific anti-skin autoantibodies in a cohort of patients with inherited epidermolysis bullosa. Orphanet J Rare Dis. 2013;8:132. 34. Kawakami Y, Kajita A, Hasui KI, Matsuda Y, Iwatsuki K, Morizane S. Elevated expression of interleukin-6 (IL-6) and serum amyloid A (SAA) in the skin and the serum of recessive dystrophic epidermolysis bullosa: Skin as a possible source of IL-6 through Toll-like receptor ligands and SAA. Exp Dermatol. 2024;33(3):e15040 35. Ambarsari CG, Palupi-Baroto R, Sinuraya FAG, Suryati E, Widyastuti E, Widhiati S. Nephropathy in a Child with Severe Recessive Dystrophic Epidermolysis Bullosa Treated with Cyclophosphamide: A Case Report. Case Rep Nephrol Dial. 2023;13(1):75-83. 36. Haghighi Javid A, Li D, Technau-Hafsi K, Has C. Interleukin-17A immune pattern across genetic acantholytic and blistering disorders. Clin Exp Dermatol. 2023;48(5):518-23. 37. Reimer-Taschenbrecker A, Hess M, Davidovic M, Hwang A, Hubner S, Hofsaess M, et al. IL-6 levels dominate the serum cytokine signature of severe epidermolysis bullosa: A prospective cohort study. J Eur Acad Dermatol Venereol. 2024. |
| --- |

**Table S5.** Increased inflammation parameters and activated pathways in dystrophic epidermolysis bullosa tissue (wounds, scars, blisters) when compared to controls.

| **Inflammation parameter or pathway** | **Reference** |
| --- | --- |
| Toll-like receptor pathway | [4] [17] |
| Jak-Stat pathway | [4] |
| TGF-β pathway | [20] |
| IL-10 | [4] |
| IL-20 | [4] |
| IL-6 | [4] [11] [16] [20] [17] |
| IL-17A and downstream S100 calcium-binding proteins | [13] |
| IL-1β | [16] |
| IL-8 | [40] |
| Th2 immunity cells / IL-31, IL-4Ra, IL-13 (+) cells | [14] |
| CCL2/MCP1 | [20] |
| CCL27 | [11] [41] |
| CCL15 | [11] |
| CXCL12 | [11] [41] |
| CCL21 | [41] |
| HMGB1 | [41] |
| TSLP | [14] |
| Periostin | [14] |
| SP (+) c-kit (+) cells positively correlated with IL-31 (+) cells and pruritus | [14] |
| NK1R (+) cells | [14] |
| TN-C (fibrosis biomarker) | [16] |
| MMP-9 | [40] |
| SAA | [17] |

IL: interleukin, SP: substance P, NK1R: neurokinin-1 receptor, TN-C: tenascin-C, MMP: matrix metalloproteinase, SAA: serum amyloid A, TGF-β: Transforming growth factor β, TSLP: serum thymic stromal lymphopoietin, CCL: Chemokine (C-C motif) ligand, CXCL: Chemokine (C-X-C motif) ligand, HMGB1: High mobility group box 1

**Table S6.** Increased systemic inflammation parameters in dystrophic epidermolysis bullosa (EB) serum when compared to controls or other EB types.

| **Inflammation parameter or pathway** | **Reference** |
| --- | --- |
| CRP, negatively correlated with weight Z-scores, bone mineral density and pubertal status | [21] [29] [42] [43] |
| IL-6, positively correlated with BEBS, wound BSA | [17] [21] [22] [23] [24] [27] [28] |
| IL-6/IL-10 ratio | [23] |
| IL-10 | [28] |
| IL-4 | [21] |
| IL-31 | [14] [21] |
| IL-1β | [27] [28] |
| IL-2 | [27] [28] |
| IgE | [21] |
| IgG | [29] |
| IgA, negatively correlated with weight Z-scores | [29] |
| Leucocyte counts | [29] |
| TSLP | [14] [21] |
| SAA | [17] [24] |
| TNF-β | [27] [28] |
| IFN-γ | [27] [28] |
| CCL11 | [11] |
| CCL15 | [11] |
| CCL21 | [41] |
| CXCL8 | [11] |
| CXCL7 | [11] |
| CXCL12, positively correlated with wound BSA | [11] [41] |
| HMGB1 | [41] |
| Active TGF-β1 | [44] |

CRP: C-reactive protein, BSA: body surface area, IL: interleukin, SAA: serum amyloid A, TGF-β: Transforming growth factor β, TSLP: serum thymic stromal lymphopoietin, TNF-β: Tumor necrosis factor beta**,** CCL: Chemokine (C-C motif) ligand, CXCL: Chemokine (C-X-C motif) ligand, IFN-γ: interferon gamma, HMGB1: High mobility group box 1, BEBS: Birmingham EB Severity score

**Table S7.** Studies that provide evidence for treatments targeting inflammation in dystrophic epidermolysis bullosa.

| **Treatment** | **Patients** | **Evidence** | **Reference** |
| --- | --- | --- | --- |
| Dupilumab | 2 patients with DEB pruriginosa | - Patients received loading dose of 600mg dupilumab, followed by 300g every 4 weeks - Treatment with dupilumab significantly improved symptoms (resolution of nodular prurigo-like lesions, decrease of Visual Analog Scale-VAS score, decrease of Children’s Dermatology Life Quality Index-CDLQI score, fewer recurrent blisters and decrease in Epidermolysis Bullosa Disease Activity and Scaring Index-BDASI score) after 4 weeks. - Eosinophil counts decreased slightly over the 20-week treatment period in the two patients. - The mean percentage of Th2 cells fell from 6.10% to 4.39%, while the mean percentage of Th17 cells increased from 1.64% to 4.71% after 20-weeks of dupilumab therapy. - Immunofluorescence staining from skin samples indicated an increase in type VII collagen levels 20 week after dupilumab administration in the two DEB patients. | [30] |
| Losartan | 7 patients with RDEB | - Losartan administrated in an oral daily dose of 0.7mg/kg for six weeks and resulted in a subjective improvement of the clinical features (number of new lesions/erosions decreased in five patients, and mucosal soreness diminished in all seven patients) - severity of the disease objectively improved based on BEBS score (30.1 ± 12.8 versus 23.3 ± 10.4, before and after treatment, p = 0.018), accompanied by improvement of quality of life, as determined by the EB-QoL questionnaire (24.0 ± 8.1 versus 17.7 ± 5.5, p = 0.018) - Histopathology of the selected lesions revealed after treatment increased number of mast cells, and enhanced microvasculature in the mid and lower dermis. - The width of collagen bundles in dermis was suggested to be decreased in four samples and changed from dense to loose in appearance, suggesting a decrease in dermal fibrosis. | [31] |
| Methotrexate and statins |  | - A transcriptomic profiling study was conducted to make in silico predictions for compounds that could reverse RDEB gene expression signatures. - The analysis identified 50 compounds that countered the signatures of dysregulated genes between normal and wounded RDEB skin, potentially promoting wound healing in RDEB . - A search was then conducted to assess evidence from in vitro or clinical studies, accessibility, and safety for the top ten compound. Four commercially available compounds were identified: simvastatin, methotrexate, anafranil, and fulvestrant. - Methotrexate emerged as the leading candidate, prompting further testing in animal models or patients with RDEB to validate the in silico predictions regarding wound healing. | [4] |
| JAK inhibitors | 12 DEB patients | - Upadacitinb and baricitinib treatments resulted in a rapid and sustained decrease in itch in weeks 2 and 4. - In 33.3% of the patients and 70% of the patients there was a decrease of at least 3 points in the pruritus VAS score from baseline at weeks 2 and 4 respectively (mean percentage changes from baseline at weeks 2 and 4 were -42.9% and -52.7% respectively). - 33.3% of the patients showed at least a 2-point reduction in pain intensity from baseline at week 4 and 66.7% of the patients showed a reduction in the number of new blisters, which was correlated with the reduction in the pruritus score. - This may indicate that pain also has an inflammatory component and that the anti-inflammatory therapy with JAK inhibitors could effectively break the vicious cycle of itch-scratch-blister formation in DEB. - At baseline, 75% of the patients had an erythema score of 2 or 3, an indicator of cutaneous inflammation. At week 4, however, only 16.7% of the patients had an erythema score of 2 or 3. - No patient discontinued treatment because of serious adverse events. | [32] |
| TNF-α inhibitors | 29-year-old woman with mild DEB and concomitant psoriatic arthritis | - subcutaneous etanercept, at a dosage of 50mg twice a week led to improvement of joint symptoms after 2 weeks of therapy - Furthermore, it resulted in a remarkable improvement of pruritus and blisters, along with a reduction in the appearance of new bullous lesions during the first 3 months of therapy - Etanercept was continued, and after 3 years, complete resolution of psoriatic arthritis with persistent good control of DEB and sporadic occurrence of new lesions were observed. | [33] |
| Systemic G-CSF | 7 patients with DEB (6 RDEB and 1 DDEB) | - 6 daily doses (10 μg/kg/dose) of systemic G-CSF. - The patients were revaluated on day 7. - There was a median reduction of 75.5% in lesional surface area size of selected wounds and 36.6% in blister/erosion counts. - Wounds that were present for more than 60 days, were greater than 50cm^2^ in surface area and had evidence of superinfection or malignancy were excluded from the assessment of the lesional surface area. - None of the patients experienced any side effects. - The results of this study need to be validated in greater patient cohorts, but the intermittent administration of G-CSF may be an adjunct therapy for persistent wounds that don’t respond to conventional local therapies. - Whether a different dosage or more frequent administration of G-CSF may be more effective, as well as its long-term effect needs also to be validated in future studies. | [34] |

BEBS: Birmingham Epidermolysis Bullosa Severity, BDASI: Epidermolysis Bullosa Disease Activity and Scarring Index, CDLQI: Children’s Dermatology Life Quality Index, DDEB: dominant dystrophic epidermolysis bullosa, DEB: dystrophic epidermolysis bullosa, EB-QoL: Epidermolysis bullosa quality of life questionnaire, G-CSF: granulocyte-colony stimulating factor, JAK: Janus kinase, RDEB: recessive dystrophic epidermolysis bullosa, TNF-a: Tumor necrosis factor alpha, VAS: Visual Analog Scale

**Table S8**. Increased anti-skin antibodies in dystrophic epidermolysis bullosa (EB) serum when compared to controls or other EB types and their correlation with systemic inflammation parameters.

| **Anti-skin antibody** | **Reference** |
| --- | --- |
| Anti-BP180   - Positively correlated with IL-6, IL-6/IL-10 ratio, disease severity, BEBS score, IFN-γ - Negatively correlated with IL-5, C7 skin expression | [23] [27] [28] [36] [37] |
| Anti-BP230   - Positively correlated with IL-6, IL-10, IL-6/IL-10 ratio, BEBS score, IFN-γ - Negatively correlated with IL-5 | [27] [28] [36] [23] |
| Anti-C7   - Positively correlated with IL-10, IFN-γ - Negatively correlated with IL-5 | [27] [28] [36] [23] |
| Anti-laminin 332 | [36] |
| Anti-DSG1 | [28] |
| Anti-DSG3 | [28] |

IL: interleukin, IFN-γ: interferon gamma, BEBS: Birmingham EB Severity score, C7: type VII collagen, DSG: desmoglein

**Table S9**. Summary statistics for each study included in the meta-analysis.

| **Inflammation Marker** | **PMID** | **Comparison** | **SMD** | **Standard Error of SMD** | **Group 1** | **n 1** | **Group 2** | **n 2** | **Year** | **Paper quality** | **Study quality** |
| --- | --- | --- | --- | --- | --- | --- | --- | --- | --- | --- | --- |
| anti-BP180 | 27669234 | H-P | -1.24555 | 0.292071 | H | 38 | P | 19 | 2016 | 3 | 3 |
| anti-BP180 | 27669234 | H-P and PH together | -1.04837 | 0.23373 | H | 38 | P and PH together | 42 | 2016 | 3 | 3 |
| anti-BP180 | 27669234 | H-PH | -0.642 | 0.267364 | H | 38 | PH | 23 | 2016 | 3 | 3 |
| anti-BP180 | 27669234 | P-P and PH together | 0.197185 | 0.276768 | P | 19 | P and PH together | 42 | 2016 | 3 | 3 |
| anti-BP180 | 27669234 | P-PH | 0.603555 | 0.312414 | P | 19 | PH | 23 | 2016 | 3 | 3 |
| anti-BP180 | 27669234 | P and PH together-PH | 0.40637 | 0.2607 | P and PH together | 42 | PH | 23 | 2016 | 3 | 3 |
| anti-BP180 | 29276186 | P-PH | 1.005925 | 0.446005 | P | 13 | PH | 10 | 2017 | 3 | 2 |
| anti-BP180 | 38389191 | P-PH | 0.950955 | 0.574581 | P | 10 | PH | 5 | 2024 | 1 | 2 |
| anti-BP180 | 24007552 | P-PH | 1.320871 | 0.437187 | P | 17 | PH | 10 | 2013 | 1 | 3 |
| anti-BP230 | 27669234 | H-P | -1.32584 | 0.293516 | H | 38 | P | 19 | 2016 | 3 | 3 |
| anti-BP230 | 27669234 | H-P and PH together | -0.95161 | 0.232027 | H | 38 | P and PH together | 42 | 2016 | 3 | 3 |
| anti-BP230 | 27669234 | H-PH | 0.1448 | 0.264348 | H | 38 | PH | 23 | 2016 | 3 | 3 |
| anti-BP230 | 27669234 | P-P and PH together | 0.374227 | 0.277516 | P | 19 | P and PH together | 42 | 2016 | 3 | 3 |
| anti-BP230 | 27669234 | P-PH | 1.47064 | 0.323996 | P | 19 | PH | 23 | 2016 | 3 | 3 |
| anti-BP230 | 27669234 | P and PH together-PH | 1.096413 | 0.268728 | P and PH together | 42 | PH | 23 | 2016 | 3 | 3 |
| anti-BP230 | 29276186 | P-PH | 1.000071 | 0.445719 | P | 13 | PH | 10 | 2017 | 3 | 2 |
| anti-BP230 | 38389191 | P-PH | 0.85238 | 0.569402 | P | 10 | PH | 5 | 2024 | 1 | 2 |
| anti-BP230 | 24007552 | P-PH | 1.35294 | 0.439 | P | 17 | PH | 10 | 2013 | 1 | 3 |
| anti-C7 | 27669234 | H-P | -1.65308 | 0.300245 | H | 38 | P | 19 | 2016 | 3 | 3 |
| anti-C7 | 27669234 | H-P and PH together | -1.40854 | 0.241364 | H | 38 | P and PH together | 42 | 2016 | 3 | 3 |
| anti-C7 | 27669234 | H-PH | -0.18385 | 0.264448 | H | 38 | PH | 23 | 2016 | 3 | 3 |
| anti-C7 | 27669234 | P-P and PH together | 0.244535 | 0.276923 | P | 19 | P and PH together | 42 | 2016 | 3 | 3 |
| anti-C7 | 27669234 | P-PH | 1.469229 | 0.32397 | P | 19 | PH | 23 | 2016 | 3 | 3 |
| anti-C7 | 27669234 | P and PH together-PH | 1.224694 | 0.270989 | P and PH together | 42 | PH | 23 | 2016 | 3 | 3 |
| anti-C7 | 29276186 | P-PH | 0.989843 | 0.445222 | P | 13 | PH | 10 | 2017 | 3 | 2 |
| anti-C7 | 38389191 | P-PH | 0.894137 | 0.571532 | P | 10 | PH | 5 | 2024 | 1 | 2 |
| anti-C7 | 24007552 | P-PH | 1.410792 | 0.442359 | P | 17 | PH | 10 | 2013 | 1 | 3 |
| anti-DSG1 | 27669234 | H-P | -1.11955 | 0.289973 | H | 38 | P | 19 | 2016 | 3 | 3 |
| anti-DSG1 | 27669234 | H-P and PH together | -0.93685 | 0.231781 | H | 38 | P and PH together | 42 | 2016 | 3 | 3 |
| anti-DSG1 | 27669234 | H-PH | -0.22775 | 0.264588 | H | 38 | PH | 23 | 2016 | 3 | 3 |
| anti-DSG1 | 27669234 | P-P and PH together | 0.182696 | 0.276727 | P | 19 | P and PH together | 42 | 2016 | 3 | 3 |
| anti-DSG1 | 27669234 | P-PH | 0.891799 | 0.315229 | P | 19 | PH | 23 | 2016 | 3 | 3 |
| anti-DSG1 | 27669234 | P and PH together-PH | 0.709104 | 0.263341 | P and PH together | 42 | PH | 23 | 2016 | 3 | 3 |
| anti-DSG3 | 27669234 | H-P | -1.49271 | 0.296781 | H | 38 | P | 19 | 2016 | 3 | 3 |
| anti-DSG3 | 27669234 | H-P and PH together | -1.26824 | 0.238154 | H | 38 | P and PH together | 42 | 2016 | 3 | 3 |
| anti-DSG3 | 27669234 | H-PH | -0.13942 | 0.264336 | H | 38 | PH | 23 | 2016 | 3 | 3 |
| anti-DSG3 | 27669234 | P-P and PH together | 0.224464 | 0.276853 | P | 19 | P and PH together | 42 | 2016 | 3 | 3 |
| anti-DSG3 | 27669234 | P-PH | 1.353285 | 0.321894 | P | 19 | PH | 23 | 2016 | 3 | 3 |
| anti-DSG3 | 27669234 | P and PH together-PH | 1.128822 | 0.269277 | P and PH together | 42 | PH | 23 | 2016 | 3 | 3 |
| IFN-γ | 27669234 | H-P | -0.03944 | 0.280987 | H | 38 | P | 19 | 2016 | 3 | 3 |
| IFN-γ | 27669234 | H-P and PH together | -0.08001 | 0.223945 | H | 38 | P and PH together | 42 | 2016 | 3 | 3 |
| IFN-γ | 27669234 | H-PH | -0.07779 | 0.264233 | H | 38 | PH | 23 | 2016 | 3 | 3 |
| IFN-γ | 27669234 | P-P and PH together | -0.04057 | 0.276492 | P | 19 | P and PH together | 42 | 2016 | 3 | 3 |
| IFN-γ | 27669234 | P-PH | -0.03835 | 0.310026 | P | 19 | PH | 23 | 2016 | 3 | 3 |
| IFN-γ | 27669234 | P and PH together-PH | 0.00222 | 0.259399 | P and PH together | 42 | PH | 23 | 2016 | 3 | 3 |
| IL-10 | 27669234 | H-P | 0.145339 | 0.28113 | H | 38 | P | 19 | 2016 | 3 | 3 |
| IL-10 | 27669234 | H-P and PH together | -0.10617 | 0.22399 | H | 38 | P and PH together | 42 | 2016 | 3 | 3 |
| IL-10 | 27669234 | H-PH | -0.20101 | 0.264499 | H | 38 | PH | 23 | 2016 | 3 | 3 |
| IL-10 | 27669234 | P-P and PH together | -0.25151 | 0.276948 | P | 19 | P and PH together | 42 | 2016 | 3 | 3 |
| IL-10 | 27669234 | P-PH | -0.34635 | 0.310808 | P | 19 | PH | 23 | 2016 | 3 | 3 |
| IL-10 | 27669234 | P and PH together-PH | -0.09483 | 0.25947 | P and PH together | 42 | PH | 23 | 2016 | 3 | 3 |
| IL-10 | 29276186 | H-P | 0.89172 | 0.377062 | H | 18 | P | 13 | 2017 | 3 | 2 |
| IL-10 | 29276186 | H-PH | -0.90554 | 0.406885 | H | 18 | PH | 10 | 2017 | 3 | 2 |
| IL-10 | 29276186 | P-PH | -1.79726 | 0.465097 | P | 13 | PH | 10 | 2017 | 3 | 2 |
| IL-10 | 38376135 | P-PH | 0.532193 | 0.747914 | P | 19 | PH | 2 | 2024 | 1 | 4 |
| IL-12 | 27669234 | H-P | 0.395572 | 0.282115 | H | 38 | P | 19 | 2016 | 3 | 3 |
| IL-12 | 27669234 | H-P and PH together | -0.3468 | 0.224985 | H | 38 | P and PH together | 42 | 2016 | 3 | 3 |
| IL-12 | 27669234 | H-PH | -0.34546 | 0.26511 | H | 38 | PH | 23 | 2016 | 3 | 3 |
| IL-12 | 27669234 | P-P and PH together | -0.74237 | 0.280535 | P | 19 | P and PH together | 42 | 2016 | 3 | 3 |
| IL-12 | 27669234 | P-PH | -0.74104 | 0.313625 | P | 19 | PH | 23 | 2016 | 3 | 3 |
| IL-12 | 27669234 | P and PH together-PH | 0.001335 | 0.259399 | P and PH together | 42 | PH | 23 | 2016 | 3 | 3 |
| IL-1β | 27669234 | H-P | -0.41532 | 0.282231 | H | 38 | P | 19 | 2016 | 3 | 3 |
| IL-1β | 27669234 | H-P and PH together | -0.16438 | 0.224134 | H | 38 | P and PH together | 42 | 2016 | 3 | 3 |
| IL-1β | 27669234 | H-PH | 0.185046 | 0.264451 | H | 38 | PH | 23 | 2016 | 3 | 3 |
| IL-1β | 27669234 | P-P and PH together | 0.250937 | 0.276946 | P | 19 | P and PH together | 42 | 2016 | 3 | 3 |
| IL-1β | 27669234 | P-PH | 0.600367 | 0.312389 | P | 19 | PH | 23 | 2016 | 3 | 3 |
| IL-1β | 27669234 | P and PH together-PH | 0.34943 | 0.260362 | P and PH together | 42 | PH | 23 | 2016 | 3 | 3 |
| IL2 | 27669234 | H-P | -0.40085 | 0.282145 | H | 38 | P | 19 | 2016 | 3 | 3 |
| IL2 | 27669234 | H-P and PH together | -0.5164 | 0.226314 | H | 38 | P and PH together | 42 | 2016 | 3 | 3 |
| IL2 | 27669234 | H-PH | -0.53454 | 0.266393 | H | 38 | PH | 23 | 2016 | 3 | 3 |
| IL2 | 27669234 | P-P and PH together | -0.11555 | 0.276579 | P | 19 | P and PH together | 42 | 2016 | 3 | 3 |
| IL2 | 27669234 | P-PH | -0.13368 | 0.310134 | P | 19 | PH | 23 | 2016 | 3 | 3 |
| IL2 | 27669234 | P and PH together-PH | -0.01813 | 0.259401 | P and PH together | 42 | PH | 23 | 2016 | 3 | 3 |
| IL-4 | 27669234 | H-P | -0.50498 | 0.282829 | H | 38 | P | 19 | 2016 | 3 | 3 |
| IL-4 | 27669234 | H-P and PH together | -0.90866 | 0.231321 | H | 38 | P and PH together | 42 | 2016 | 3 | 3 |
| IL-4 | 27669234 | H-PH | -0.93081 | 0.270823 | H | 38 | PH | 23 | 2016 | 3 | 3 |
| IL-4 | 27669234 | P-P and PH together | -0.40368 | 0.277685 | P | 19 | P and PH together | 42 | 2016 | 3 | 3 |
| IL-4 | 27669234 | P-PH | -0.42583 | 0.311212 | P | 19 | PH | 23 | 2016 | 3 | 3 |
| IL-4 | 27669234 | P and PH together-PH | -0.02215 | 0.259403 | P and PH together | 42 | PH | 23 | 2016 | 3 | 3 |
| IL-4 | 38376135 | P-PH | 0.832052 | 0.563633 | P | 19 | PH | 4 | 2024 | 1 | 4 |
| IL-6 | 27669234 | H-P | 0.059554 | 0.281002 | H | 38 | P | 19 | 2016 | 3 | 3 |
| IL-6 | 27669234 | H-P and PH together | -0.04827 | 0.223908 | H | 38 | P and PH together | 42 | 2016 | 3 | 3 |
| IL-6 | 27669234 | H-PH | -0.04823 | 0.264204 | H | 38 | PH | 23 | 2016 | 3 | 3 |
| IL-6 | 27669234 | P-P and PH together | -0.10782 | 0.276566 | P | 19 | P and PH together | 42 | 2016 | 3 | 3 |
| IL-6 | 27669234 | P-PH | -0.10778 | 0.310093 | P | 19 | PH | 23 | 2016 | 3 | 3 |
| IL-6 | 27669234 | P and PH together-PH | 4.23E-05 | 0.259399 | P and PH together | 42 | PH | 23 | 2016 | 3 | 3 |
| IL-6 | 29276186 | H-P | -2.51467 | 0.457816 | H | 18 | P | 13 | 2017 | 3 | 2 |
| IL-6 | 29276186 | H-PH | 0.07757 | 0.394498 | H | 18 | PH | 10 | 2017 | 3 | 2 |
| IL-6 | 29276186 | P-PH | 2.592241 | 0.508794 | P | 13 | PH | 10 | 2017 | 3 | 2 |
| IL-6 | 38376135 | P-PH | 0.358213 | 0.280569 | P | 40 | PH | 19 | 2024 | 1 | 4 |
| IL-8 | 27669234 | H-P | 1.2846 | 0.292764 | H | 38 | P | 19 | 2016 | 3 | 3 |
| IL-8 | 27669234 | H-P and PH together | 0.771214 | 0.229266 | H | 38 | P and PH together | 42 | 2016 | 3 | 3 |
| IL-8 | 27669234 | H-PH | 0.762637 | 0.268659 | H | 38 | PH | 23 | 2016 | 3 | 3 |
| IL-8 | 27669234 | P-P and PH together | -0.51339 | 0.278426 | P | 19 | P and PH together | 42 | 2016 | 3 | 3 |
| IL-8 | 27669234 | P-PH | -0.52196 | 0.311812 | P | 19 | PH | 23 | 2016 | 3 | 3 |
| IL-8 | 27669234 | P and PH together-PH | -0.00858 | 0.259399 | P and PH together | 42 | PH | 23 | 2016 | 3 | 3 |
| TNF-a | 27669234 | H-P | -1.08103 | 0.289373 | H | 38 | P | 19 | 2016 | 3 | 3 |
| TNF-a | 27669234 | H-P and PH together | -1.0381 | 0.233542 | H | 38 | P and PH together | 42 | 2016 | 3 | 3 |
| TNF-a | 27669234 | H-PH | -0.00667 | 0.264186 | H | 38 | PH | 23 | 2016 | 3 | 3 |
| TNF-a | 27669234 | P-P and PH together | 0.04293 | 0.276494 | P | 19 | P and PH together | 42 | 2016 | 3 | 3 |
| TNF-a | 27669234 | P-PH | 1.074362 | 0.317554 | P | 19 | PH | 23 | 2016 | 3 | 3 |
| TNF-a | 27669234 | P and PH together-PH | 1.031432 | 0.267671 | P and PH together | 42 | PH | 23 | 2016 | 3 | 3 |
| TNF-a | 38376135 | P-PH | 0.386223 | 0.553059 | P | 19 | PH | 4 | 2024 | 1 | 4 |
| TNF-β | 27669234 | H-P | -1.21602 | 0.291561 | H | 38 | P | 19 | 2016 | 3 | 3 |
| TNF-β | 27669234 | H-P and PH together | -1.18198 | 0.236328 | H | 38 | P and PH together | 42 | 2016 | 3 | 3 |
| TNF-β | 27669234 | H-PH | -1.20589 | 0.275234 | H | 38 | PH | 23 | 2016 | 3 | 3 |
| TNF-β | 27669234 | P-P and PH together | 0.034037 | 0.276488 | P | 19 | P and PH together | 42 | 2016 | 3 | 3 |
| TNF-β | 27669234 | P-PH | 0.010121 | 0.310017 | P | 19 | PH | 23 | 2016 | 3 | 3 |
| TNF-β | 27669234 | P and PH together-PH | -0.02392 | 0.259403 | P and PH together | 42 | PH | 23 | 2016 | 3 | 3 |
| CCL19 | 28094098 | H-PH | 0.287215 | 0.32882 | H | 13 | PH | 33 | 2017 | 3 | 2 |
| CCL21 | 28094098 | H-PH | 1.271012 | 0.306729 | H | 19 | PH | 37 | 2017 | 3 | 2 |
| CCL27 | 28094098 | H-PH | -0.25948 | 0.283302 | H | 19 | PH | 37 | 2017 | 3 | 2 |
| CCL28 | 28094098 | H-PH | -0.02129 | 0.311407 | H | 15 | PH | 33 | 2017 | 3 | 2 |
| CXCL12 | 28094098 | H-PH | -0.85052 | 0.321652 | H | 15 | PH | 34 | 2017 | 3 | 2 |
| HMGB1 | 28094098 | H-PH | -0.63811 | 0.293742 | H | 18 | PH | 37 | 2017 | 3 | 2 |
| Serum albumin | 31487386 | P-PH | -0.8281 | 0.17703 | P | 157 | PH | 43 | 2020 | 1 | 4 |
| Serum albumin | 37389358 | P-PH | 0.670117 | 0.64286 | P | 7 | PH | 4 | 2023 | 1 | 1 |
| Serum albumin | 35379269 | P-PH | -0.96071 | 0.177128 | P | 108 | PH | 52 | 2022 | 1 | 3 |
| Serum albumin | 20609141 | P-PH | 0.376008 | 0.767061 | P | 12 | PH | 2 | 2010 | 1 | 3 |
| CRP | 31487386 | P-PH | 0.765774 | 0.176327 | P | 157 | PH | 43 | 2020 | 1 | 4 |
| CRP | 35379269 | P-PH | 0.634618 | 0.172478 | P | 108 | PH | 52 | 2022 | 1 | 3 |
| CRP | 36067143 | P-PH | 2.349353 | 0.570842 | P | 12 | PH | 9 | 2022 | 1 | 3 |
| CRP | 38376135 | P-PH | 0.373097 | 0.319829 | P | 34 | PH | 14 | 2024 | 1 | 4 |
| HB | 31487386 | P-PH | -0.60516 | 0.174759 | P | 157 | PH | 43 | 2020 | 1 | 4 |
| HB | 37389358 | P-PH | -0.28625 | 0.588448 | P | 7 | PH | 5 | 2023 | 1 | 1 |
| HB | 35379269 | P-PH | -0.72534 | 0.173592 | P | 108 | PH | 52 | 2022 | 1 | 3 |
| HB | 20609141 | P-PH | -0.14573 | 0.76002 | P | 13 | PH | 2 | 2010 | 1 | 3 |
| IgA | 31487386 | P-PH | 0.498104 | 0.173912 | P | 157 | PH | 43 | 2020 | 1 | 4 |
| IgA | 35379269 | P-PH | 0.201166 | 0.169164 | P | 108 | PH | 52 | 2022 | 1 | 3 |
| IgG | 31487386 | P-PH | 0.478666 | 0.173776 | P | 157 | PH | 43 | 2020 | 1 | 4 |
| IgG | 35379269 | P-PH | 0.745766 | 0.173862 | P | 108 | PH | 52 | 2022 | 1 | 3 |
| IgM | 31487386 | P-PH | -0.30505 | 0.172794 | P | 157 | PH | 43 | 2020 | 1 | 4 |
| IgM | 35379269 | P-PH | 0.558997 | 0.171658 | P | 108 | PH | 52 | 2022 | 1 | 3 |
| IGE | 38376135 | P-PH | 0.556434 | 0.55358 | P | 20 | PH | 4 | 2024 | 1 | 4 |
| TGF-β | 38376135 | P-PH | -0.28527 | 0.494906 | P | 23 | PH | 5 | 2024 | 1 | 4 |
| TSLP | 38376135 | P-PH | 0.597331 | 0.801773 | P | 8 | PH | 2 | 2024 | 1 | 4 |
| Leukocytes | 31487386 | P-PH | 0.480555 | 0.173789 | P | 157 | PH | 43 | 2020 | 1 | 4 |
| Creatinine | 35379269 | P-PH | -0.83762 | 0.175164 | P | 108 | PH | 52 | 2022 | 1 | 3 |

SMD: Standardized Mean Difference, n: sample size, HB: hemoglobin, CRP: C-reactive protein, anti-C7: type VII collagen autoantibodies, anti-BP180: anti-BP180 autoantibodies, anti-BP230: anti-BP230 autoantibodies, anti-DSG1: anti-desmoglein 1 autoantibodies, anti-DSG3: anti-desmoglein 3 autoantibodies, IFN-γ: interferon gamma, IgG: immunoglobulin G, IgM: immunoglobulin M, IgA: immunoglobulin A, IgE: immunoglobulin E, TNF-a: tumor necrosis factor alpha, TNF-β: Tumor necrosis factor beta, IL: interleukin, TGF-β: Transforming growth factor β, TSLP: serum thymic stromal lymphopoietin, P: patients with dystrophic epidermolysis bullosa, PH: patients with other types of epidermolysis bullosa, H: healthy controls. Paper quality: 0= expert opinion, 1=observational study, 2=diagnostic study, 3=case-control study, 4=cohort study, 5=randomized controlled trial, 6=Meta-analysis. Study quality (Joanna Briggs Institute Critical Appraisal Checklist for Cohort Studies): 1=very low, 2=low, 3=moderate, 4=high

**Table S10**. Medians of the 8 inflammatory markers from single studies reporting medians. For these studies only descriptive summaries were possible.

| **Inflammation**  **Marker** | **Unit** | **Comparator**  **Group** | **Median** | **Minimum** | **Maximum** | **PMID** |
| --- | --- | --- | --- | --- | --- | --- |
| anti-DSG1 | U/mL | H | 2.12 | 0 | 15.09 | 27669234 |
| anti-DSG1 | U/mL | P | 5.62 | 1.25 | 38.83 | 27669234 |
| anti-DSG1 | U/mL | P and PH together | 3.83 | 0.18 | 38.83 | 27669234 |
| anti-DSG1 | U/mL | PH | 2.67 | 0.18 | 20.2 | 27669234 |
| anti-DSG3 | U/mL | H | 1.58 | 0.08 | 8.38 | 27669234 |
| anti-DSG3 | U/mL | P | 6.14 | 0.94 | 40.4 | 27669234 |
| anti-DSG3 | U/mL | P and PH together | 3.72 | 0 | 40.4 | 27669234 |
| anti-DSG3 | U/mL | PH | 2.8 | 0 | 10.02 | 27669234 |
| IFN-γ | pg/mL | H | 176.34 | 73.56 | 4910 | 27669234 |
| IFN-γ | pg/mL | P | 360.25 | 110.6 | 4607.18 | 27669234 |
| IFN-γ | pg/mL | P and PH together | 330.16 | 79.18 | 5000 | 27669234 |
| IFN-γ | pg/mL | PH | 279.12 | 79.18 | 5000 | 27669234 |
| IL-12 | pg/mL | H | 1.36 | 1 | 7.29 | 27669234 |
| IL-12 | pg/mL | P | 1.65 | 1.22 | 3.8 | 27669234 |
| IL-12 | pg/mL | P and PH together | 1.52 | 1.14 | 9.21 | 27669234 |
| IL-12 | pg/mL | PH | 1.44 | 1.14 | 9.21 | 27669234 |
| IL-1B | pg/mL | H | 2.75 | 0.88 | 189.66 | 27669234 |
| IL-1B | pg/mL | P | 37.74 | 2.23 | 192.67 | 27669234 |
| IL-1B | pg/mL | P and PH together | 16.62 | 1.16 | 192.67 | 27669234 |
| IL-1B | pg/mL | PH | 8.03 | 1.16 | 144.31 | 27669234 |
| IL-2 | pg/mL | H | 31.61 | 8.01 | 2400 | 27669234 |
| IL-2 | pg/mL | P | 120.7 | 34.86 | 3475.02 | 27669234 |
| IL-2 | pg/mL | P and PH together | 111.23 | 18.76 | 3992.66 | 27669234 |
| IL-2 | pg/mL | PH | 105.15 | 18.76 | 3992.66 | 27669234 |
| IL-8 | pg/mL | H | 27.96 | 12.96 | 939.06 | 27669234 |
| IL-8 | pg/mL | P | 49.7 | 21.97 | 124.24 | 27669234 |
| IL-8 | pg/mL | P and PH together | 45.09 | 13.14 | 449 | 27669234 |
| IL-8 | pg/mL | PH | 44 | 13.14 | 449 | 27669234 |
| TNF-β | pg/mL | H | 1267.11 | 802.84 | 3764.79 | 27669234 |
| TNF-β | pg/mL | P | 1719.14 | 1149.51 | 36000 | 27669234 |
| TNF-β | pg/mL | P and PH together | 1737.07 | 1101.8 | 36000 | 27669234 |
| TNF-β | pg/mL | PH | 1753.29 | 1101.8 | 36000 | 27669234 |

anti-DSG1: anti-desmoglein 1 autoantibodies, anti-DSG3: anti-desmoglein 3 autoantibodies, IFN-γ: interferon gamma, IL: interleukin, TNF-β: Tumor necrosis factor beta, P: patients with dystrophic epidermolysis bullosa, PH: patients with other types of epidermolysis bullosa, H: healthy controls

**Table S11.** Standardized mean differences for the 13 inflammatory markers with data from multiple studies.

| **Marker** | **Reference**  **Group** | **Comparator**  **Group** | **Number of available**  **studies** | **Mean**  **source** | $\boldsymbol{\tau}$**^2^** | **I^2^** | **Standardized**  **Mean**  **Difference** | **95% CI** | **95% PI** |
| --- | --- | --- | --- | --- | --- | --- | --- | --- | --- |
| IL-6 | P | H | 3 | median mean | 1.64 | 0.89 | -1.03 | (-2.79; 0.74) | (18.88; 0.9) |
| IL-6 | P | P and PH together | 3 | median mean | 1.64 | 0.89 | -0.59 | (-2.88; 1.69) | (21.43; 1.17) |
| IL-6 | P | PH | 3 | median mean | 1.64 | 0.89 | -0.89 | (-2.41; 0.62) | (18.12; 0.77) |
| TNF-a | P | H | 2 | median mean | 0.03 | 0.14 | -0.97 | (-1.61; -0.34) |  |
| TNF-a | P | P and PH together | 2 | median mean | 0.03 | 0.14 | 0.06 | (-0.56; 0.68) |  |
| TNF-a | P | PH | 2 | median mean | 0.03 | 0.14 | -0.88 | (-1.49; -0.27) |  |
| IL-10 | P | H | 3 | median mean | 0.53 | 0.69 | 0.31 | (-0.76; 1.39) | (11.91; 0.55) |
| IL-10 | P | P and PH together | 3 | median mean | 0.53 | 0.69 | 0.41 | (-0.96; 1.78) | (13.24; 0.7) |
| IL-10 | P | PH | 3 | median mean | 0.53 | 0.69 | 0.64 | (-0.36; 1.64) | (11.96; 0.51) |
| IL-4 | P | H | 2 | median mean | 0.58 | 0.74 | -0.78 | (-2.28; 0.73) |  |
| IL-4 | P | P and PH together | 2 | median mean | 0.58 | 0.74 | 0.13 | (-1.37; 1.63) |  |
| IL-4 | P | PH | 2 | median mean | 0.58 | 0.74 | -0.12 | (-1.34; 1.11) |  |
| serum albumin | P | PH | 4 | mean | 0.39 | 0.64 | 0.43 | (-0.31; 1.17) | (3.57; 0.38) |
| CRP | P | PH | 4 | mean | 0.34 | 0.69 | -0.89 | (-1.54; -0.24) | (1.99; 0.33) |
| HB | P | PH | 4 | mean | <0.0001 | 0.00 | 0.64 | (0.4; 0.87) | (1.15; 0.12) |
| IgA | P | PH | 2 | mean | 0.01 | 0.33 | -0.35 | (-0.64; -0.06) |  |
| IgG | P | PH | 2 | mean | 0.01 | 0.15 | -0.61 | (-0.87; -0.35) |  |
| IgM | P | PH | 2 | mean | 0.34 | 0.92 | -0.13 | (-0.97; 0.72) |  |
| anti-BP180 | P | H | 4 | median mean | <0.0001 | 0.00 | -1.41 | (-1.92; -0.9) | (-0.29; 0.26) |
| anti-BP180 | P | P and PH together | 4 | median mean | <0.0001 | 0.00 | -0.36 | (-0.84; 0.13) | (0.7; 0.25) |
| anti-BP180 | P | PH | 4 | median mean | <0.0001 | 0.00 | -0.89 | (-1.3; -0.49) | (-0.01; 0.21) |
| anti-BP230 | P | H | 4 | median mean | <0.0001 | 0.00 | -1.20 | (-1.7; -0.7) | (-0.1; 0.26) |
| anti- BP230 | P | P and PH together | 4 | median mean | <0.0001 | 0.00 | -0.26 | (-0.74; 0.22) | (0.79; 0.25) |
| anti-BP230 | P | PH | 4 | median mean | <0.0001 | 0.00 | -1.26 | (-1.67; -0.85) | (-0.36; 0.21) |
| anti-C7 | P | H | 4 | median mean | <0.0001 | 0.00 | -1.54 | (-2.05; -1.02) | (-0.41; 0.26) |
| anti-C7 | P | P and PH together | 4 | median mean | <0.0001 | 0.00 | -0.14 | (-0.63; 0.34) | (0.92; 0.25) |
| anti-C7 | P | PH | 4 | median mean | <0.0001 | 0.00 | -1.27 | (-1.68; -0.86) | (-0.37; 0.21) |

HB: hemoglobin, CRP: C-reactive protein, anti-C7: type VII collagen autoantibodies, anti-BP180: anti-BP180 autoantibodies, anti-BP230: anti-BP230 autoantibodies, IgG: immunoglobulin G, IgM: immunoglobulin M, IgA: immunoglobulin A, TNF-a: tumor necrosis factor alpha, IL-4: interleukin 4, IL-6: interleukin 6, IL-10: interleukin 10, CI: confidence interval.

Summary Estimates describe standardized mean differences between comparators and reference groups in inflammatory markers with multiple evidence sources, estimated using random-effects network meta-analysis models implemented via the netmeta package. Networks were constructed differently than the conventional approach of NMA; we considered nodes to be patient groups: P: patients with dystrophic epidermolysis bullosa, PH: patients with other types of epidermolysis bullosa, H: healthy controls. The initial protocol planned to analyze continuous outcomes as mean differences. However, initial analysis revealed differences in the underlying scales of raw scores and medians. To standardize values, they were converted to Cohen’s d and analyzed as standardized mean differences. We used the REML estimator to calculate network-wide between-study heterogeneity (τ²), assuming common heterogeneity across comparisons. 95% prediction intervals (PI) were generated for comparisons involving more than two studies.

**Table S12.** Summary of limitations of existing studies for tissue and systemic inflammation in dystrophic epidermolysis bullosa (DEB) and proposals for future studies.

| **Limitations of existing studies** |
| --- |
| - Heterogenous patient groups due to the rare nature of the disease: various epidermolysis bullosa types and DEB subtypes (e.g. localized, intermediate, severe, inversa, pruriginosa) are very frequently investigated as one patient group - Different types of *COL7A1* mutations are pooled together - No stratification according to age, disease stage/complications, type/evolution of the skin lesions - Only observational, retrospective, descriptive studies, case reports and small case series - No prospective large-scale studies, due to their complex expensive design and long duration - No large interventional double-blinded placebo-controlled studies (ethical considerations for the placebo group) - No multicentric studies (e.g. bias due to medical care, economic status etc.) |
| **Proposals for future studies** |
| - Randomized controlled studies to compare the efficacy and safety of drugs targeting important inflammation cytokines, as well as downstream signaling components or dual-agent approaches - Longitudinal studies to investigate the impact of bacterial colonization composition and susceptibility on wound resolution, with or without topical therapies - Longitudinal studies to investigate the natural course of the disease and inflammation: At what time point does inflammation cause permanent damage to internal organs and irreversible systemic extracutaneous complications? - Studies to investigate the pathogenicity of autoimmune bullous diseases anti-skin autoantibodies and whether DEB patients could benefit from immunosuppressive drugs to reduce pruritus and blister formation - Studies that would investigate weather additional anti-inflammatory therapies, along with gene therapy, could provide wound healing benefits to DEB patients - Use of AI modeling tools to study complex inflammatory pathways, as well as produce missing variables, in order to generate longitudinal data that may provide important information for the natural course of the disease |
