# Supplementary material for: Tissue and systemic inflammation in dystrophic epidermolysis bullosa: a systematic review and meta-analysis
Source: Orphanet J Rare Dis. 2025 Sep 23;20:479. doi: 10.1186/s13023-025-04034-2 (PMC12455789; doi:10.1186/s13023-025-04034-2)

**Supplementary Figures**

**Figure S1**. Flowchart of systematic literature review, extracted meta-analysis outcomes, studies and patient groups. EB: epidermolysis bullosa.


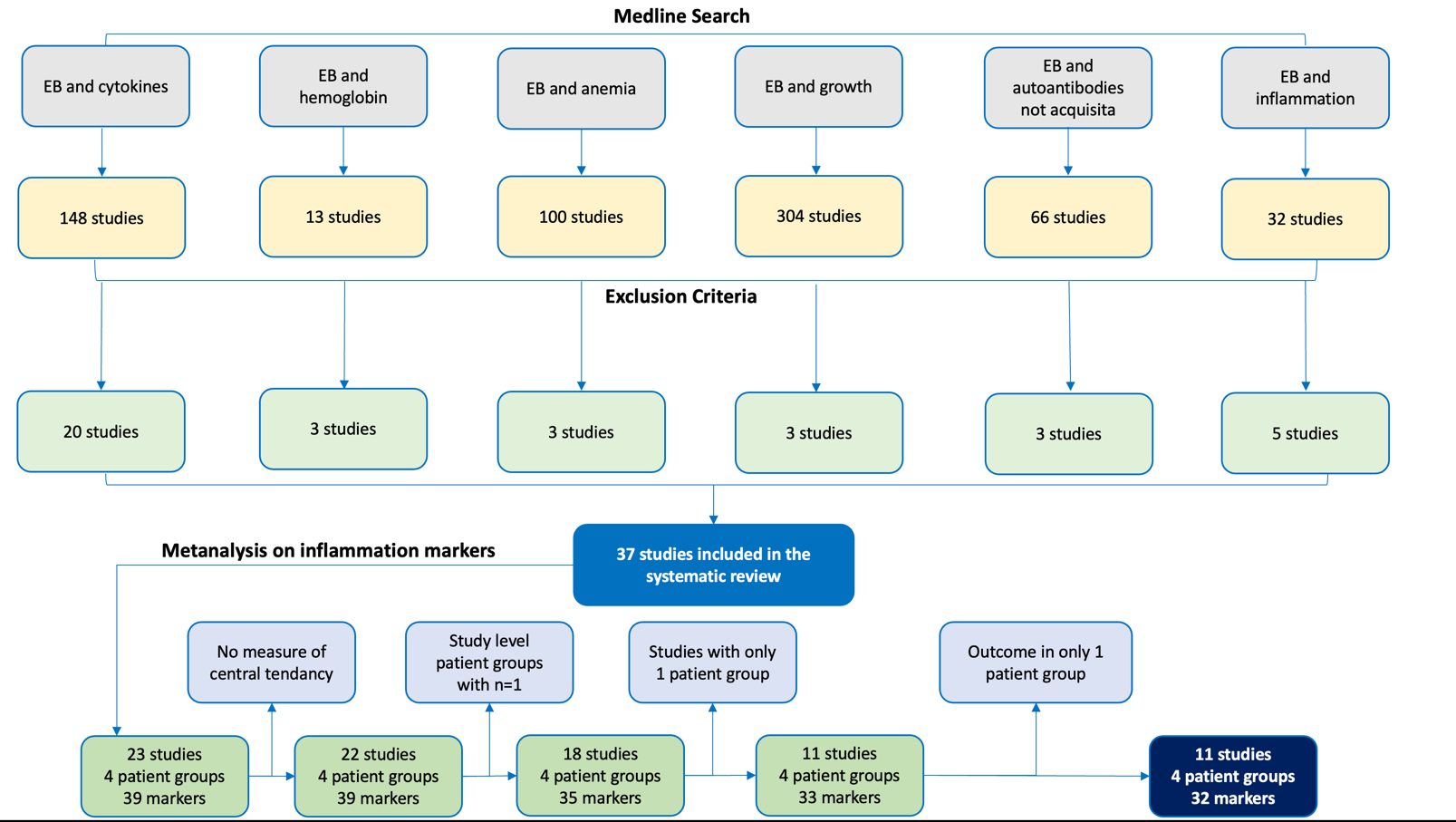


**Figure S2**. Flowchart of analysis strategies


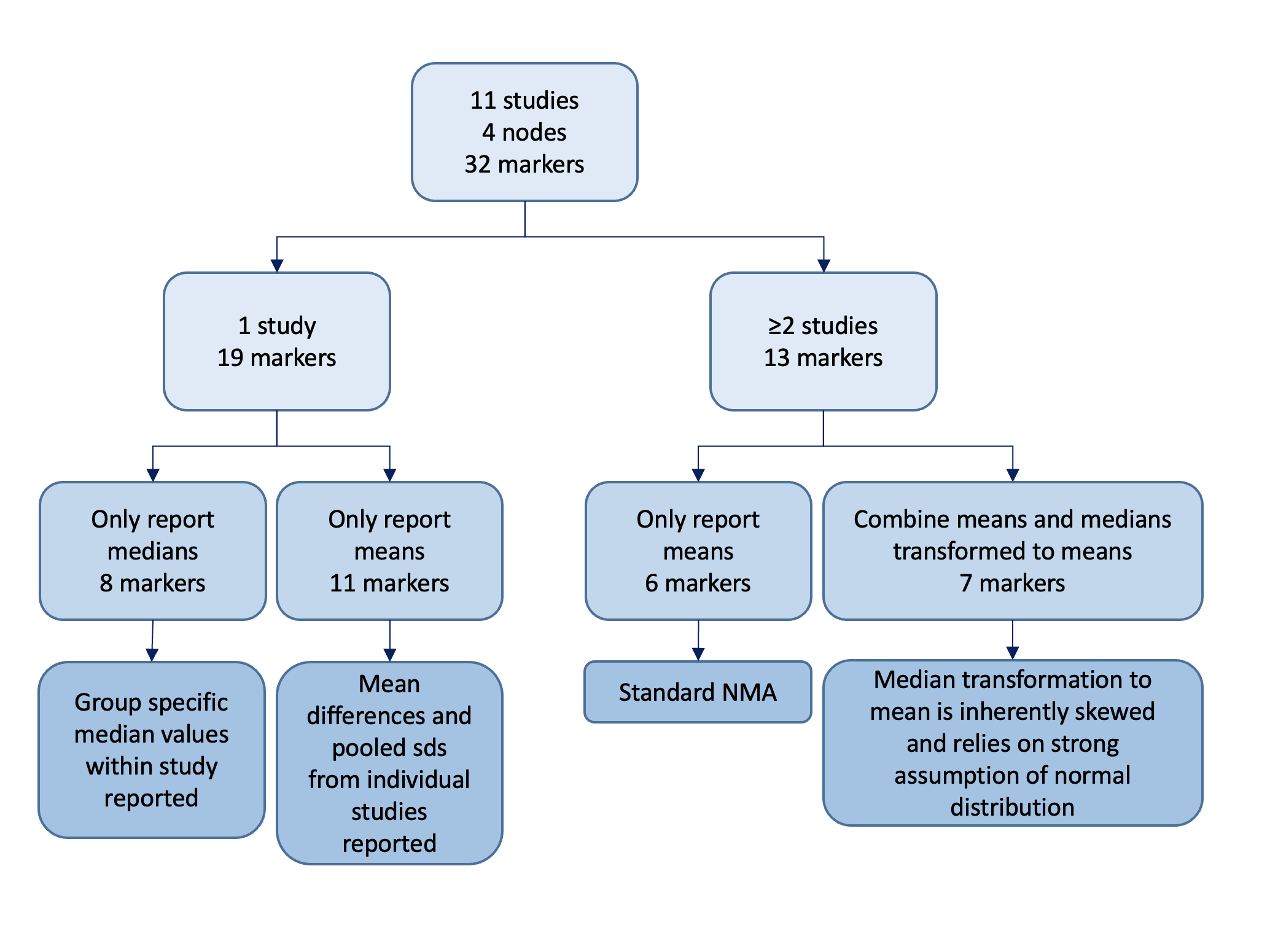


**Figure S3**. Network geometry for available quantitative evidence. The network geometry shows the observed available inflammatory markers and connections of studies containing the available patient groups. Pairwise comparisons were conducted between healthy controls (H) and patients with other types of epidermolysis bullosa (PH), as well as patients with dystrophic epidermolysis bullosa (P) and PH. Multi-group comparisons were conducted among H, P, and PH. HB: hemoglobin, CRP: C-reactive protein, C7: collagen type VII autoantibodies, BP180: anti-BP180 autoantibodies, BP230: anti-BP230 autoantibodies, IgG: immunoglobulin G, IgM: immunoglobulin M, IgA: immunoglobulin A, TNF-a: tumor necrosis factor alpha, IL-4: interleukin 4, IL-6: interleukin 6, IL-10: interleukin 10, TGF-β: Transforming growth factor β, TSLP: serum thymic stromal lymphopoietin, P: patients with dystrophic epidermolysis bullosa, PH: patients with other forms of epidermolysis bullosa, H: healthy controls.


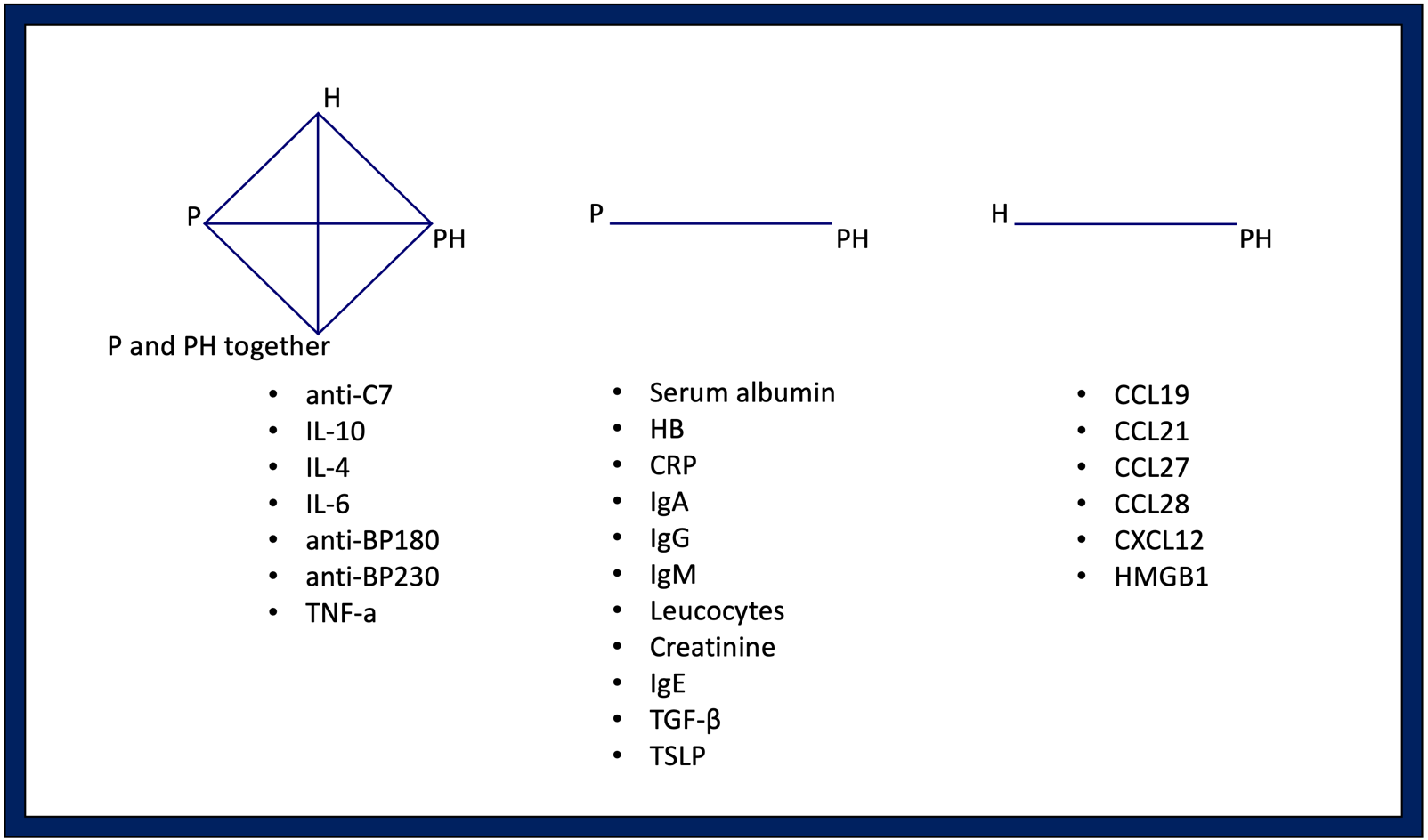

Supplement: Supplementary file 2 — Additional file 2. [file 13023_2025_4034_MOESM2_ESM.docx]
